# Simvastatin and downstream inhibitors circumvent constitutive and stromal cell-induced resistance to doxorubicin in IGHV unmutated CLL cells

**DOI:** 10.18632/oncotarget.4006

**Published:** 2015-05-27

**Authors:** Micol Rigoni, Chiara Riganti, Candida Vitale, Valentina Griggio, Ivana Campia, Marta Robino, Myriam Foglietta, Barbara Castella, Patrizia Sciancalepore, Ilaria Buondonno, Daniela Drandi, Marco Ladetto, Mario Boccadoro, Massimo Massaia, Marta Coscia

**Affiliations:** ^1^ Division of Hematology, Azienda Ospedaliero Universitaria Città della Salute e della Scienza di Torino, University of Torino, Torino, Italy; ^2^ Center for Experimental Research and Medical Studies, Azienda Ospedaliero Universitaria Città della Salute e della Scienza di Torino, Torino, Italy; ^3^ Department of Oncology, University of Torino, Torino, Italy; ^4^ S.C. Ematologia e Terapie Cellulari, Azienda Ospedaliera Ordine Mauriziano di Torino, Torino, Italy

**Keywords:** chronic lymphocytic leukemia, multidrug resistance, mevalonate pathway, statins

## Abstract

The immunoglobulin heavy-chain variable region (IGHV) mutational status is a strong determinant of remission duration in chronic lymphocytic leukemia (CLL). The aim of this work was to compare the multidrug resistance (MDR) signature of IGHV mutated and unmutated CLL cells, identifying biochemical and molecular targets potentially amenable to therapeutic intervention.

We found that the mevalonate pathway-dependent Ras/ERK1–2 and RhoA/RhoA kinase signaling cascades, and the downstream HIF-1α/P-glycoprotein axis were more active in IGHV unmutated than in mutated cells, leading to a constitutive protection from doxorubicin-induced cytotoxicity. The constitutive MDR phenotype of IGHV unmutated cells was partially dependent on B cell receptor signaling, as shown by the inhibitory effect exerted by ibrutinib. Stromal cells further protected IGHV unmutated cells from doxorubicin by upregulating Ras/ERK1–2, RhoA/RhoA kinase, Akt, HIF-1α and P-glycoprotein activities. Mevalonate pathway inhibition with simvastatin abrogated these signaling pathways and reversed the resistance of IGHV unmutated cells to doxorubicin, also counteracting the protective effect exerted by stromal cells. Similar results were obtained via the targeted inhibition of the downstream molecules ERK1–2, RhoA kinase and HIF-1α.

Therefore, targeting the mevalonate pathway and its downstream signaling cascades is a promising strategy to circumvent the MDR signature of IGHV unmutated CLL cells.

## INTRODUCTION

Chronic lymphocytic leukemia (CLL) is characterized by a highly heterogeneous clinical course and a poor curability with conventional chemotherapy treatment approaches. The immunoglobulin heavy-chain variable region (IGHV) mutational status has emerged as a powerful prognosticator: patients with IGHV unmutated (UM) CLL cells display inferior survival rates [1, 2] and shorter complete remission duration than IGHV mutated (M) patients [3].

Stromal cells (SCs) are known to protect CLL cells from spontaneous apoptosis and drug-induced cytotoxicity. We previously reported that IGHV UM cells are more dependent than M cells on microenvironment-mediated signals for their survival [4]. However, whether IGHV M and UM CLL cells differ for their *ex vivo* susceptibility to chemotherapy is controversial [5, 6].

Results from clinical trials have shown that fludarabine, even when used as a single agent, induced higher remission rates than other chemotherapies, such as CAP (cyclophosphamide, doxorubicin, prednisone) or CHOP (cyclophosphamide, doxorubicin, vincristine, prednisone), in previously untreated CLL patients [7, 8]. However, the reasons accounting for the lower effectiveness of anthracycline-containing regimens in CLL remain largely unexplored.

One of the main mechanisms of chemoresistance is the overexpression of membrane transporters which actively extrude chemotherapy drugs, a process called multidrug resistance (MDR). Anthracyclines, such as doxorubicin (Doxo), are substrates of one of the best characterized drug efflux pump, the P-glycoprotein (Pgp/ABCB1), which is encoded by the MDR1 gene [9].

Pgp activity is directly related to the amount of cell cholesterol in the plasma membrane [10], and its expression is regulated by the transcription factor hypoxia-inducible factor-1 alpha (HIF-1α), whose activation is dependent on Ras/ERK1–2 and RhoA/RhoA kinase signaling pathways [11].

All these pathways are under the control of the mevalonate (Mev) pathway, a highly conserved metabolic cascade which produces sterols, such as cholesterol, and isoprenoids, such as farnesyl pyrophosphate (FPP) and geranylgeranyl pyrophosphate (GGPP). The latter are necessary for the isoprenylation of Ras and RhoA GTPases, and for the activation of their downstream signaling pathways [12].

The Mev pathway can be pharmacologically inhibited using statins (e.g. simvastatin, SIM) or aminobisphosphonates (e.g. zoledronic acid, ZA) [13], and we have already shown that ZA can restore the sensitivity of MDR positive (MDR+) solid tumor cell lines to Doxo [14].

CLL cells carrying IGHV UM genes have significantly higher levels of Mev pathway activity, which are thought amenable to pharmacological manipulation by SIM and ZA [15]. It is currently unknown whether the higher activity of the Mev pathway in IGHV UM cells translates into a MDR+ phenotype, and whether the targeted inhibition of the Mev pathway or downstream signaling can eventually counteract the MDR+ signature of CLL cells.

The aim of this study was twofold: 1) to characterize the MDR status of IGHV M and UM cells, by evaluating the activity of Ras/ERK1–2, RhoA/RhoA kinases, and HIF-1α/Pgp axis under basal conditions and after exposure to SCs; 2) to determine whether targeting the Mev pathway and its downstream signaling eventually restores the sensitivity of MDR+ CLL cells to Doxo.

## RESULTS

### The Ras/ERK1–2 and RhoA/RhoA kinase signaling pathways and the HIF-1α/Pgp axis are more active in IGHV UM than M CLL cells

**Figure 1:**
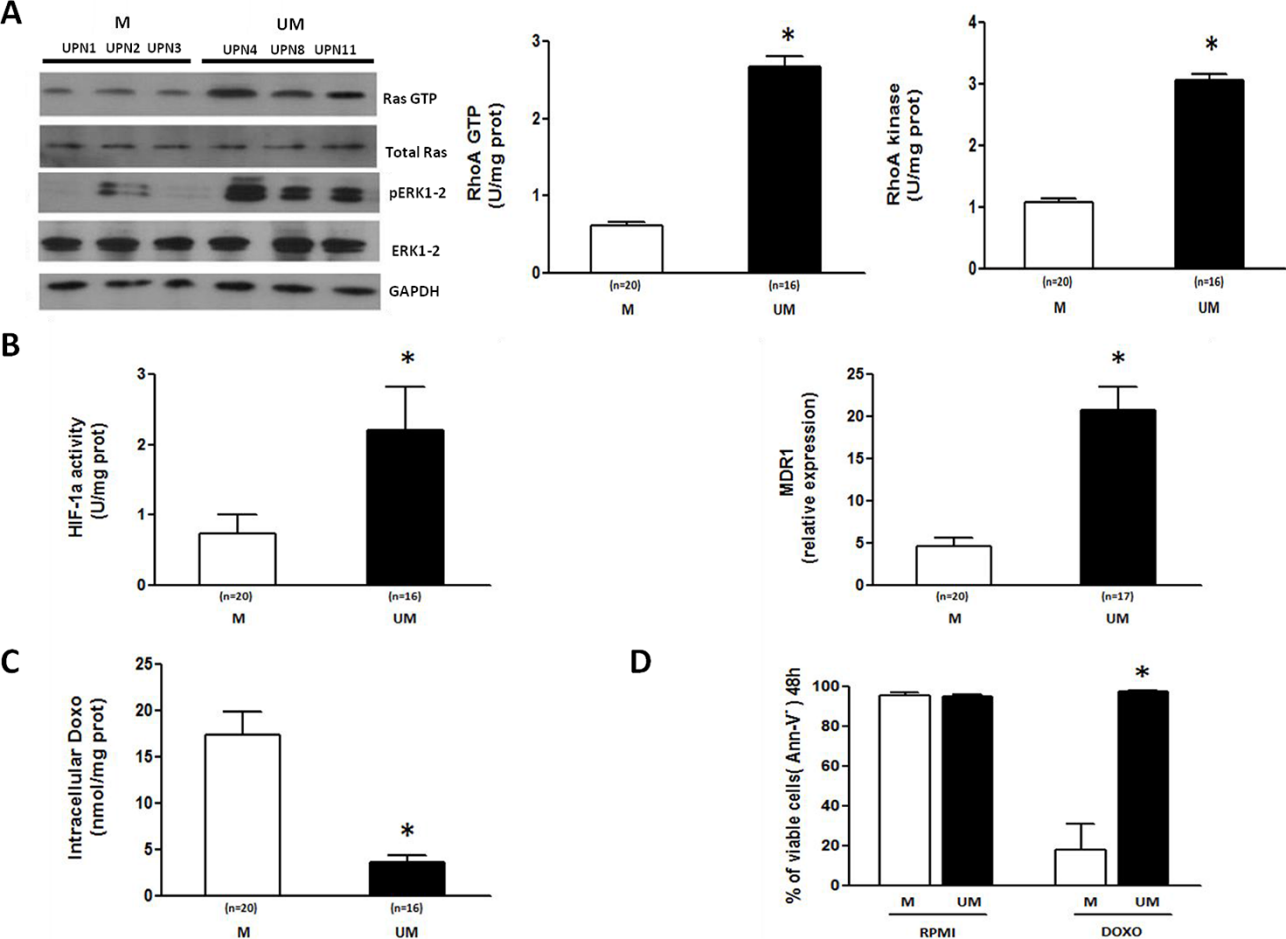
The Ras/ERK1–2 and RhoA/RhoA kinase signaling pathways and the HIF-1α/Pgp axis are more active in IGHV UM than M CLL cells The activity of the Ras/ERK1–2 and RhoA/RhoA kinase signaling cascades and the HIF-1α/Pgp axis were measured in CLL cells isolated from the peripheral blood of IGHV M and UM patients after 24-hour *ex vivo* culture. **A.** Ras and ERK1–2 kinase activities were measured by Western Blot (WB) (left side). IGHV UM cells have a higher expression of the active forms of Ras (Ras GTP) and ERK1–2 (pERK1–2), than IGHV M cells. Results are from 3 representative experiments for both M and UM patients (UPN, unique patient number). RhoA GTP and RhoA Kinase activities were measured by ELISA assays (right side). IGHV UM cells have higher activities of RhoA GTP and RhoA Kinase compared to M cells (*p = 0.001). **B.** HIF-1α activity and MDR1 mRNA expression. IGHV UM cells have higher HIF-1α activity compared to M cells (*p = 0.002) after 24 hours of culture. At the same time-point, significantly higher MDR1 levels were observed in IGHV UM cells than in M cells (*p = 0.001). **C.** Intracellular Doxo accumulation. Significantly lower concentrations of Doxo were detected after 48-hour *ex vivo* exposure in IGHV UM than in M cells (*p = 0.001). **D.** Viability of CD19+/CD5+ cells was determined by Annexin-V (Ann-V) staining and cytofluorimetric analysis after 48-hour Doxo exposure. IGHV UM cells showed higher levels of viability than M cells (*p = 0.001). Results are from 7 experiments for both M and UM patients. In all panels, bars represent mean values ± SEM.

The activity of Ras- and RhoA-dependent signaling pathways was analyzed in IGHV M and UM CLL cells (>90% pure as described below) after *ex vivo* culture for 24 hours. Both type of cells exhibited detectable amounts of non-isoprenylated cytosolic Ras and unphosphorylated ERK1–2, but only IGHV UM cells showed high intracellular levels of the Ras GTP-bound active form and the Ras-downstream effector kinase phospho-ERK1–2 (Figure 1A, left), in keeping with their accelerated Mev pathway activity [15]. Similarly, the amount of active GTP-bound RhoA and the activity of the downstream RhoA kinase were significantly higher in IGHV UM than M cells (*p* always = 0.001) (Figure 1A, right).

Both ERK1–2 and RhoA kinase phosphorylate and activate the transcription factor HIF-1α [16, 17]. Accordingly, the activation of Ras/ERK1–2 and RhoA/RhoA kinase signalling pathways in IGHV UM cells led to the phosphorylation of HIF-1α (Supplementary Figure S1) and to the increase of its transcriptional activity, as shown by the significantly higher amounts of nuclear HIF-1α bound to its specific DNA target sequence (*p* = 0.002) (Figure 1B, left). As a consequence, IGHV UM cells showed higher MDR1 mRNA expression (Figure 1B, right) and lower Doxo accumulation than IGHV M cells (*p* always = 0.001) (Figure 1C).

In agreement with our previous data [4], both untreated IGHV M and UM cells showed high levels of viability after *ex vivo* culture for 48 hours, whereas Doxo treatment induced a significant decrease of viability in IGHV M compared to UM CLL cells (*p* = 0.001) (Figure 1D and Supplementary Figure S2).

### The constitutive MDR phenotype of IGHV UM cells depends on BCR signaling and is further upregulated by SC-mediated extrinsic signals

**Figure 2:**
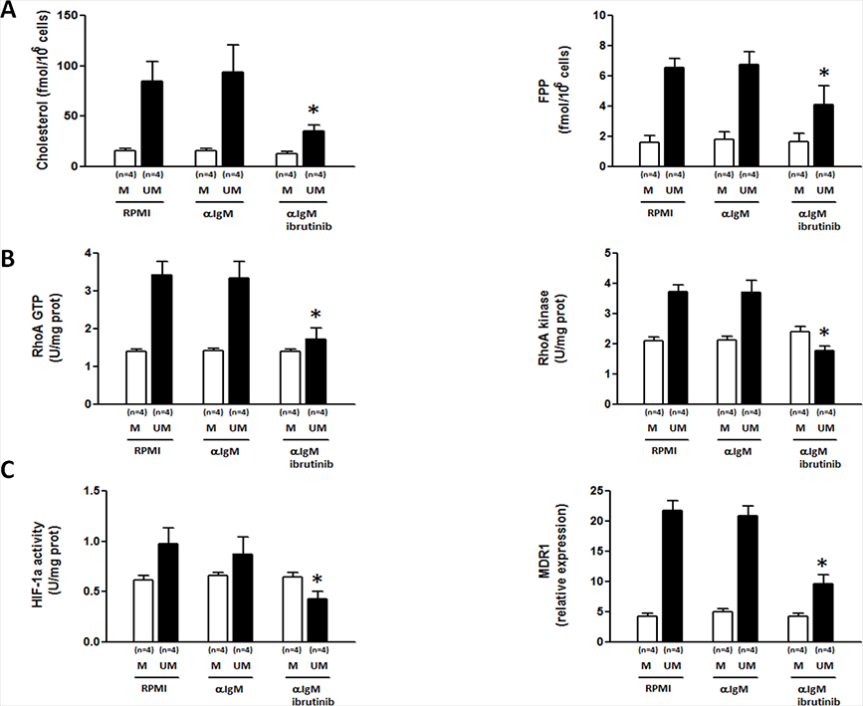
Ibrutinib inhibits the Mev pathway activity, the RhoA/RhoA kinase cascade and the HIF-1α/Pgp axis in IGHV UM but not in IGHV M cells IGHV M and UM cells were stimulated for 24 hours with anti-IgM (10 μg/well) in the absence or presence of 30-minute pre-incubation with ibrutinib (1 μM). **A.** Cholesterol and FPP production. Anti-IgM stimulation did not increase cholesterol and FPP production in IGHV M and UM cells. IgM+ibrutinib significantly reduced the levels of cholesterol and FPP in IGHV UM cells (*p = 0.04 and *p = 0.05, respectively). **B.** RhoA and RhoA kinase activity. Anti-IgM stimulation had no effect on RhoA-GTP expression and RhoA kinase activity in IGHV M and UM cells. The amount of GTP-bound RhoA and the activity of the RhoA kinase were significantly reduced by IgM+ibrutinib in IGHV UM cells (*p = 0.0015 and *p = 0.0021, respectively), but not in IGHV M cells. **C.** HIF-1α activity and MDR1 expression. Anti-IgM stimulation did not increase HIF-1α activity and MDR1 expression in IGHV M and UM cells. The activity of HIF-1α and MDR1 expression were significantly reduced by IgM+ibrutinib in IGHV UM cells (*p = 0.01 and *p = 0.0012, respectively), but not in IGHV M cells. In all panels, bars represent mean values ± SEM of side-by-side experiments performed in replicates.

IGHV UM cells display an increased signaling through the B cell receptor (BCR) compared to IGHV M cells [18]. To study the effect of BCR signaling on Mev-regulated pathways and MDR phenotype we incubated IGHV M and UM cells with anti-IgM antibody, in the presence or absence of the Bruton tyrosine kinase (BTK) inhibitor ibrutinib. Anti-IgM-mediated BCR stimulation did not increase the baseline production of cholesterol and FPP (Figure 2A), the amount of GTP-bound RhoA and the activity of RhoA kinase (Figure 2B), the transcriptional activity of HIF-1α and the expression of its target gene MDR1 (Figure 2C) in both IGHV M and UM cells. Of note, ibrutinib-mediated BCR inhibition significantly decreased the constitutively higher levels of cholesterol and FPP production, RhoA and RhoA kinase activity, HIF-1α transcriptional activity and MDR1 expression in IGHV UM cells (*p* always ≤ 0.05), whereas it did not affect the Mev pathway and Mev-regulated signaling in IGHV M cells.

**Figure 3:**
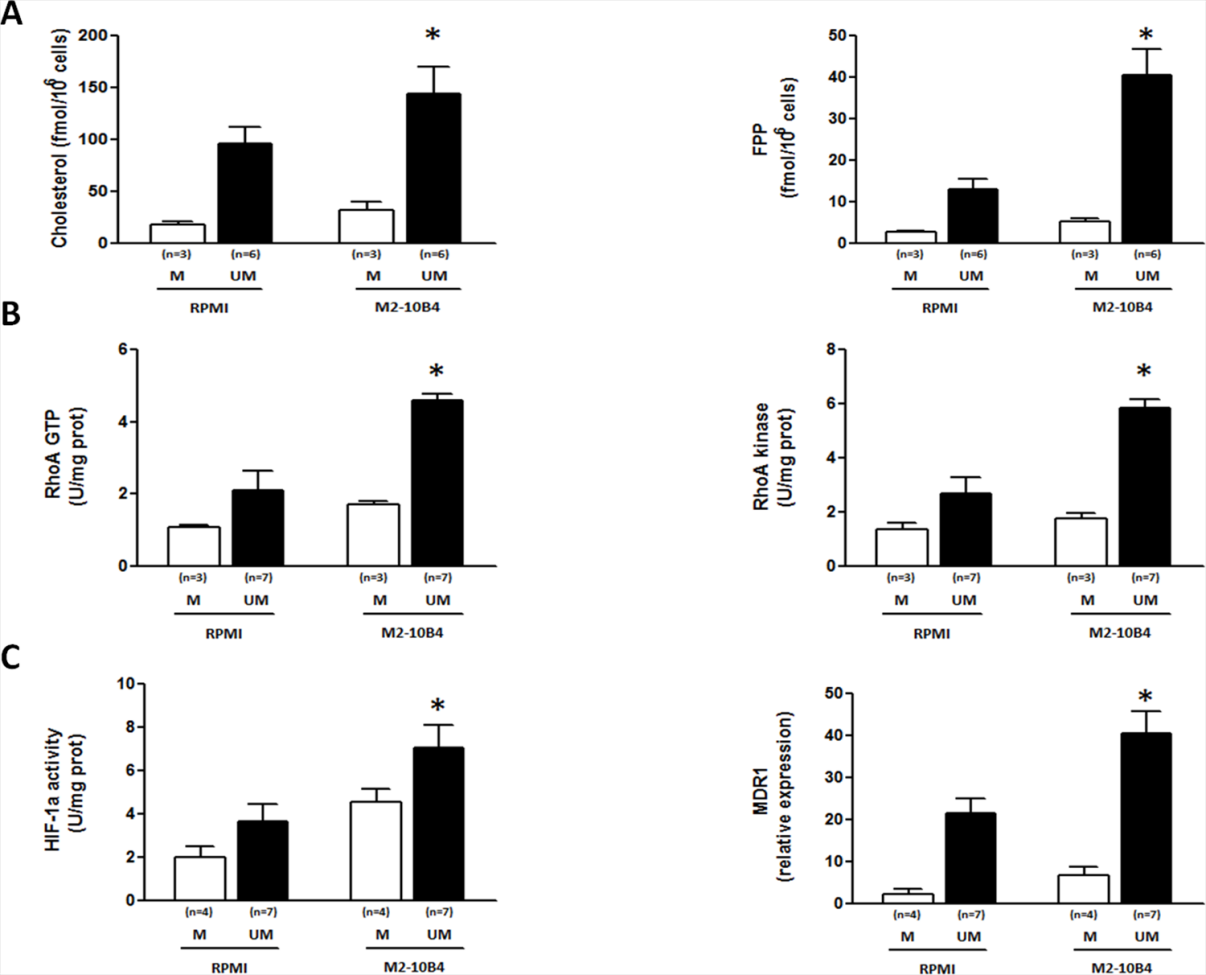
SCs upregulate the Mev pathway activity, the RhoA/RhoA kinase cascade and the HIF-1α/Pgp axis in IGHV UM but not in IGHV M CLL cells The production of cholesterol and FPP **A.** the amount of GTP-bound RhoA and the activity of the RhoA kinase **B.** the transcriptional activity of HIF-1α and the expression of the MDR1 gene **C.** were higher in IGHV UM cells cultured for 24 hours with M2–10B4 SCs compared to IGHV UM cells cultured alone (*p always ≤ 0.002). SCs did not have a significant impact on the activity of the Mev pathway and downstream molecules in IGHV M cells. In all panels, bars represent mean values ± SEM of side-by-side experiments performed in replicates.

SCs are known to confer a chemoresistant phenotype to CLL cells [19–21]. Therefore, we also sought whether IGHV M and UM cells co-cultured with the murine stromal cell line M2–10B4 upregulated the Mev pathway and the RhoA/RhoA kinase signaling pathways. IGHV M and UM cells showed high and comparable levels of cell viability after 24-hour *ex vivo* culture, both in the presence and in the absence of SCs (data not shown). After exposure to M2–10B4 SCs, the production of cholesterol and FPP (Figure 3A), the amount of GTP-bound RhoA and the activity of RhoA kinase (Figure 3B), the transcriptional activity of HIF-1α, and the expression of its target gene MDR1 (Figure 3C) were significantly increased in IGHV UM but not in M CLL cells (*p* always ≤ 0.03).

### SIM effectively reverses the MDR phenotype of IGHV UM cells and restores Doxo-induced cytotoxicity

We next examined whether SIM, which switches off the Mev pathway downstream to the rate-controlling enzyme 3-hydroxy-3-methyl-glutaryl-CoA reductase, was effective in reversing the MDR phenotype of IGHV UM cells.

**Figure 4:**
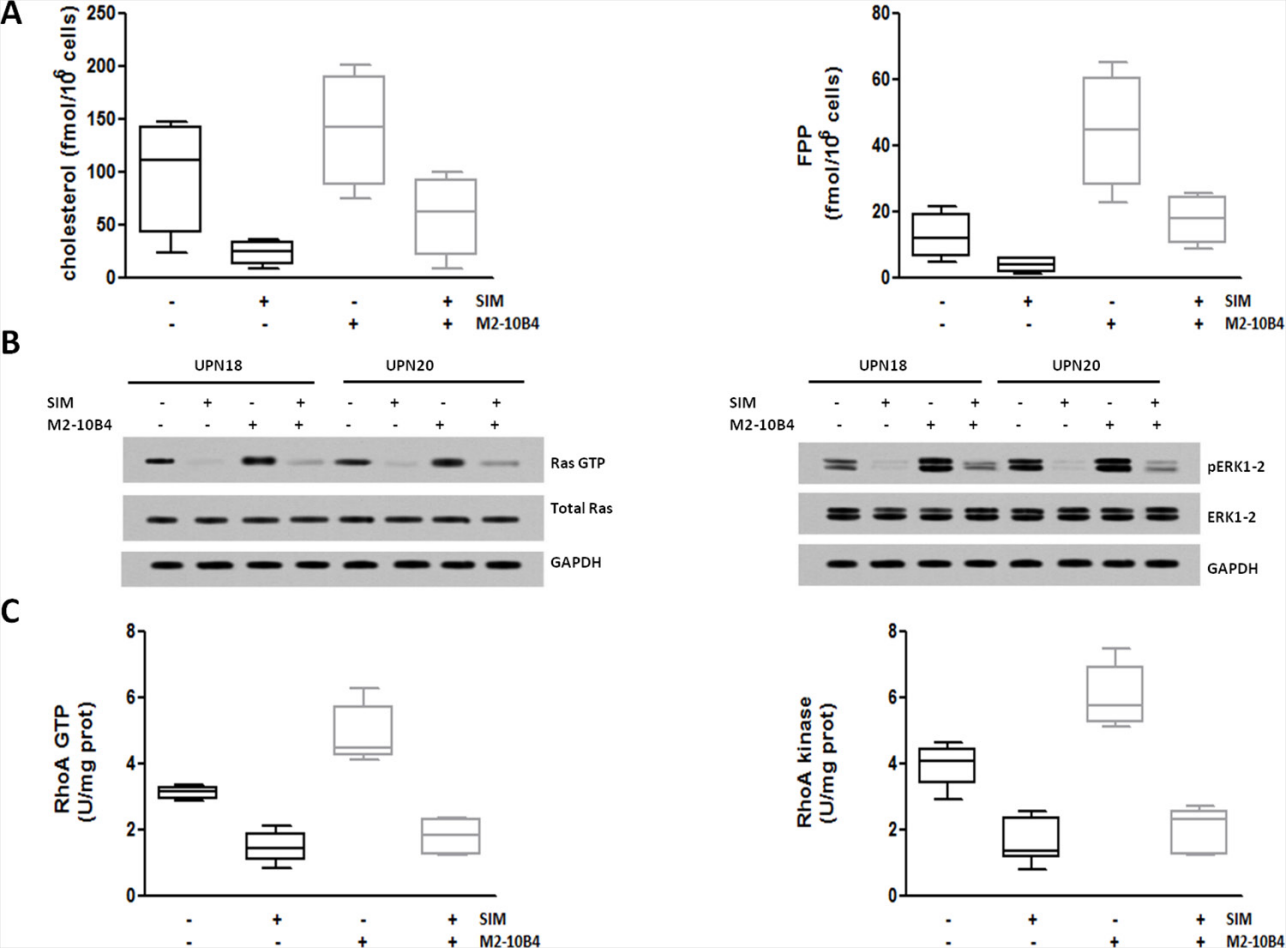
SIM-mediated inhibition of Ras/ERK1–2 and RhoA/RhoA kinase signaling pathways IGHV UM cells, cultured alone or in the presence of the murine stromal cell line M2–10B4, were exposed to 1 μM SIM. **A.** Cholesterol and FPP production. The amount of cholesterol and FPP produced by IGHV UM cells was significantly increased after 24-hour co-culture with SCs (*p* = 0.007 and *p* = 0.009, respectively). SIM significantly reduced the levels of cholesterol and FPP in IGHV UM cells both in the absence (*p* = 0.04 and *p* = 0.05, respectively) and in the presence (*p* = 0.03 and *p* = 0.01, respectively) of M2–10B4 SCs. **B.** Ras and ERK1–2 kinase activity. Co-culture with M2–10B4 SCs increased the levels of Ras-GTP and phospho-ERK1–2 kinase. SIM reduced the expression of the active forms of Ras and phospho-ERK1–2 kinase in IGHV UM cells, both in the absence and in the presence of SCs. Results are from two representative experiments (UPN, unique patient number). **C.** RhoA and RhoA kinase activity. Twenty four-hour co-culture with M2–10B4 SCs significantly increased the levels of expression of RhoA GTP and RhoA Kinase (*p* = 0.0005 and *p* = 0.0024, respectively). The expression of RhoA-GTP and RhoA kinase was significantly reduced by SIM, both in the absence (*p* < 0.0001 and *p* < 0.0001, respectively) and in the presence (*p* < 0.0001 and *p* < 0.0001, respectively) of SCs. In panels A and C results are from 8 side-by-side experiments. Box and whiskers plots represent median values, first and third quartiles, and minimum and maximum values for each dataset.

In IGHV UM cells, SIM significantly reduced cholesterol and FPP production (*p* always ≤ 0.05) (Figure 4A), the amounts of GTP-bound Ras and phospho-ERK1–2 kinases (Figure 4B), the amounts of GTP-bound RhoA and the activity of RhoA kinase (Figure 4C), also abrogating the upregulating effect exerted by M2–10B4 SCs (*p* always ≤ 0.0005).

**Figure 5:**
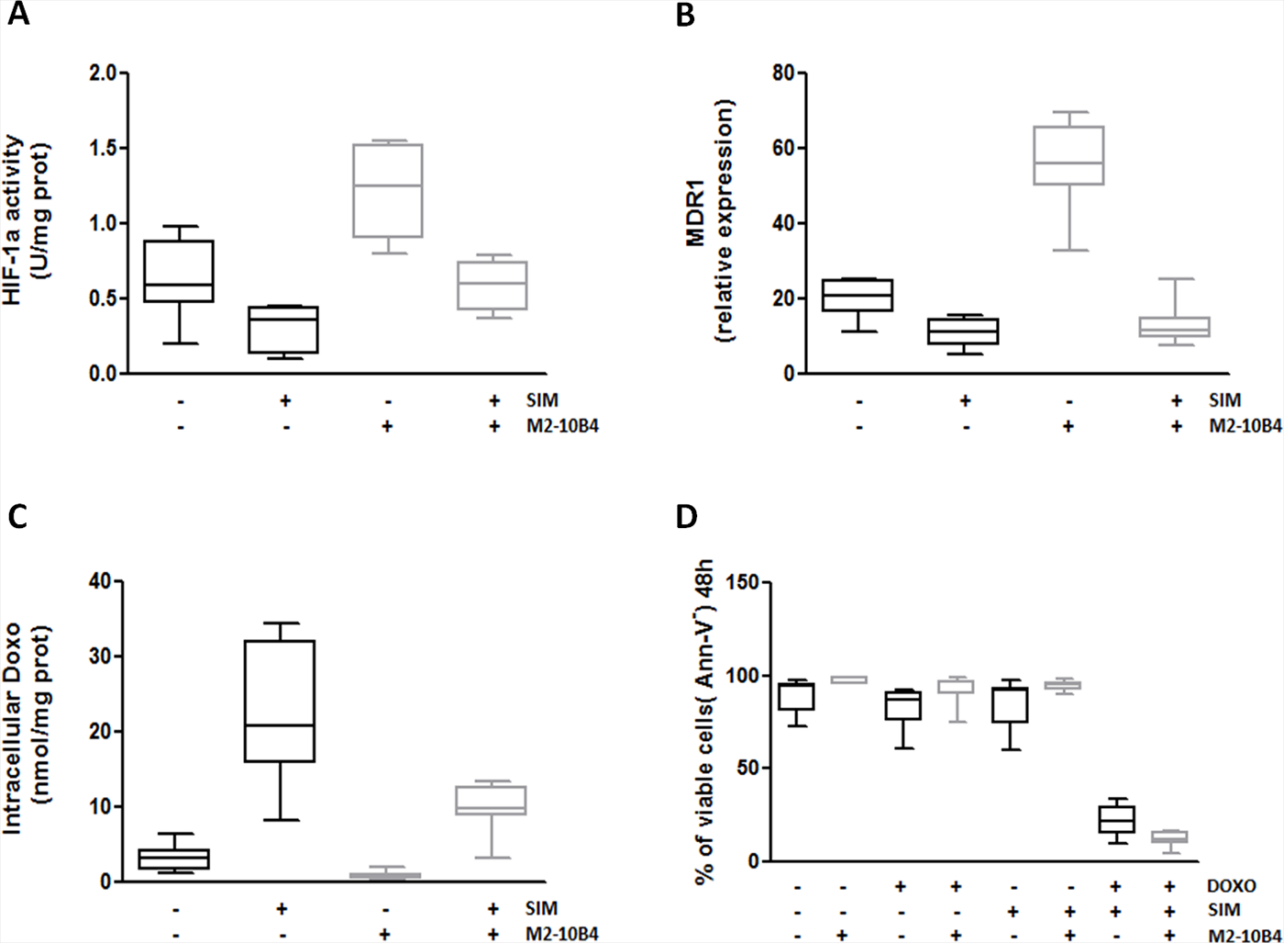
SIM-mediated inhibition of HIF-1α activity and MDR1 gene expression improves Doxo-induced cytotoxicity IGHV UM cells, cultured alone or in the presence of the murine stromal cell line M2–10B4, were exposed to 1 μM SIM. **A.** HIF-1α activity. The activity of HIF-1α was significantly increased by 24-hour co-culture with M2–10B4 SCs (*p* = 0.0007). SIM significantly reduced HIF-1α activity in IGHV UM cells, both in the absence (*p* = 0.003) and in the presence (*p* < 0.0001) of M2–10B4 SCs. **B.** MDR1 expression. Co-culture with M2–10B4 SCs significantly increased the levels of expression of MDR1 in IGHV UM cells (*p* < 0.0001). After 24-hour exposure to SIM, a significant decrease in MDR1 expression was observed in IGHV UM cells, both in the absence (*p* < 0.0001) and in the presence (*p* < 0.0001) of SCs. **C.** Doxo intracellular accumulation. The amount of intracellular Doxo in IGHV UM cells was significantly reduced by co-culture with M2–10B4 SCs (*p* = 0.010). SIM significantly increased 48-hour Doxo accumulation in IGHV UM cells cultured alone (*p* = 0.0026) or in the presence of SCs (*p* = 0.0002). **D.** Percentage of CD19+/CD5+ viable cells. IGHV UM cells were exposed to 1 μM Doxo, 1 μM SIM, and the combination SIM+Doxo for 48 hours, both in the absence and in the presence of M2–10B4 SCs. Cell viability was determined by Ann-V staining and cytofluorimetric analysis on CD19+/CD5+ cells. No decrease in viability was observed when IGHV UM cells were exposed to SIM and Doxo used alone (91% ± 2% and 90% ± 1% of CD19+/CD5+ viable cells, respectively). By contrast, a significant reduction in cell viability was induced by the combination SIM+Doxo, both in the absence (19, 5% ± 7% CD19+/CD5+ viable cells, *p* < 0.0001) and in the presence (10% ± 3% CD19+/CD5+ viable cells, *p* < 0.0001) of SCs. In panels A-D results are from 8 side-by-side experiments. Box and whiskers plot represent median values, first and third quartiles, and minimum and maximum values for each dataset.

This SIM-mediated inhibition of the Mev pathway and downstream signaling pathways was paralleled by a lower HIF-1α phosphorylation (Supplementary Figure S3) and a statistically significant decrease in HIF-1α activity and MDR1 expression in IGHV UM cells, both in the presence or absence of SCs (*p* always < 0.003) (Figure 5A, 5B). Thus, SIM significantly increased the accumulation of intracellular Doxo, even in the presence of SCs (*p* always ≤ 0.002) (Figure 5C). The SIM-induced increase in Doxo accumulation determined an enhanced cytotoxic death of IGHV UM cells after exposure to the combination SIM+Doxo for 48 hours (*p* ≤ 0.0001). Interestingly, SIM was also effective in reversing the protective effect exerted by M2–10B4 SCs (*p* ≤ 0.0001) (Figure 5D and Supplementary Figure S4).

### SIM effectively abrogates SC-induced Akt upregulation in IGHV UM cells

We next examined the effect of another Mev pathway inhibitor, ZA, which switches off the pathway downstream to FPP-synthase. ZA significantly abrogated cholesterol and FPP synthesis in IGHV UM cells cultured alone (*p* ≤ 0.05), and also in IGHV UM cells cultured with M2–10B4 SCs (*p* ≤ 0.03) (Supplementary Figure S5A, S5B). Moreover, ZA was effective in reducing the amount of the active GTP-bound form of Ras and phospho-ERK1–2, even in the presence of the M2–10B4 SCs (Supplementary Figure S5C, S5D). Similarly, the exposure of IGHV UM cells to ZA significantly reduced the amount of the active GTP-bound form of RhoA and the activity of the downstream RhoA kinase, irrespective of the presence or absence of SCs (*p* always ≤ 0.01) (Supplementary Figure S5E, S5F).

The inhibition of Ras/ERK1–2 and RhoA/RhoA kinase signaling was paralleled by a reduction of HIF-1α phosphorylation (Supplementary Figure S6), and a significant decrease in HIF-1α activity and MDR1 expression in the presence or absence of SCs (*p* always ≤ 0.05) (Supplementary Figure S7A, S7B).

Unlike SIM, ZA was unable to increase Doxo accumulation and Doxo-induced cytotoxicity in IGHV UM cells cultured in the presence or in the absence of M2–10B4 SCs (Supplementary Figure S7C, S7D).

**Figure 6:**
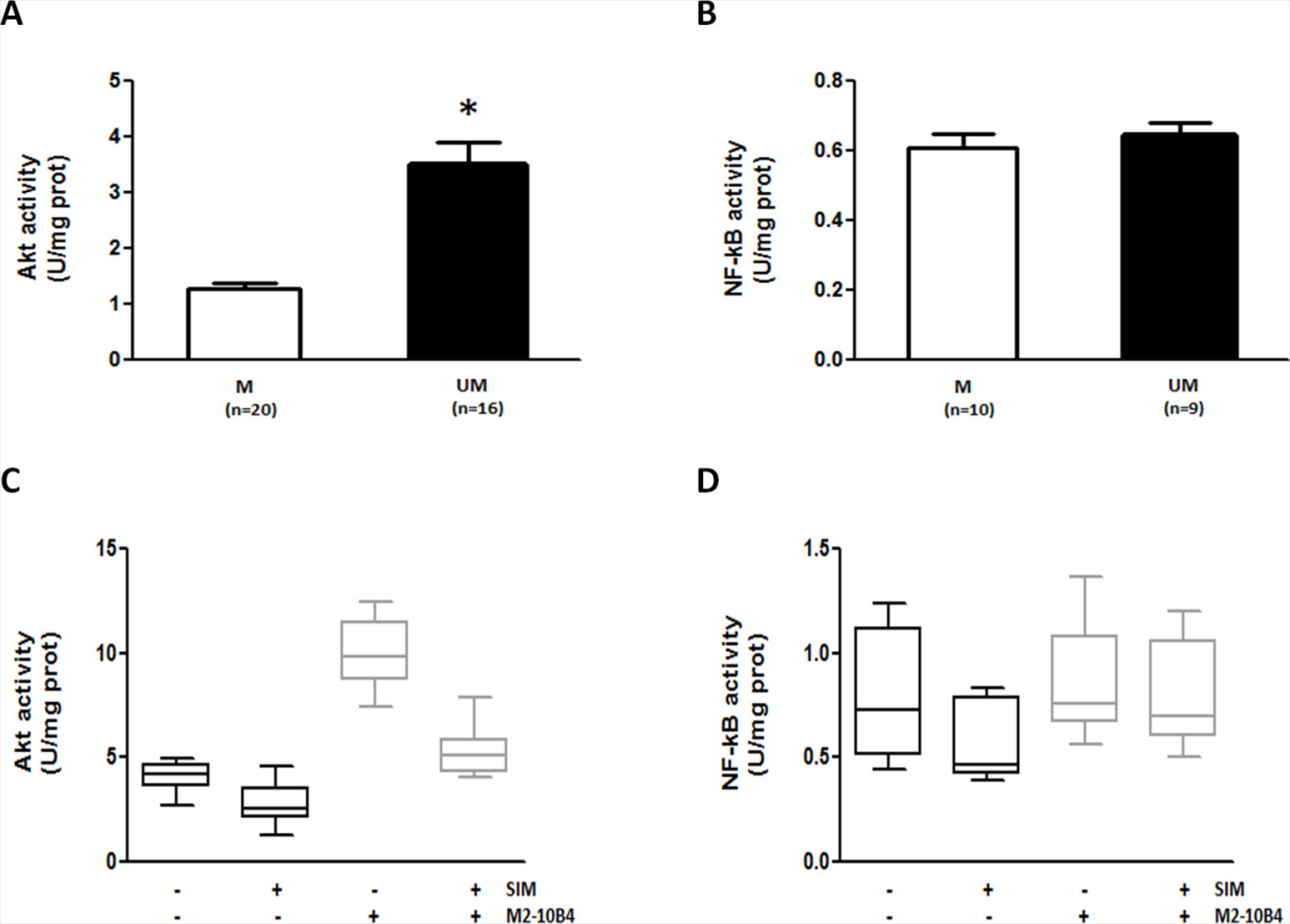
SIM effectively counteracts SC-induced Akt upregulation in IGHV UM cells IGHV UM cells had constitutive higher levels of Akt activity (*p* < 0.001) compared to IGHV M cells **A.** By contrast, there was no difference in baseline NF-kB activity between IGHV M and UM cells **B.** The Akt activity was further upregulated by the co-culture of IGHV UM cells with M2–10B4 SCs (*p* < 0.0001). SIM reduced baseline levels of Akt activity (*p* = 0.0082), and significantly counteracted SC-induced Akt upregulation (*p* = 0.0004) **C.** NF-kB activity was not upregulated by SCs, but it was significantly reduced by SIM exposure, both in the absence (*p* = 0.02) or presence of SCs (*p* = 0.034) **D.** In panels A and B bars represent mean values ± SEM. In panels C and D results are from 8 side-by-side experiments, and box and whiskers plot represent median values, first and third quartiles, and minimum and maximum values for each dataset.

To further elucidate the difference between ZA and SIM, we also evaluated the effects on Akt and NF-kB, which are well-known pro-survival factors for CLL cells [22]. The baseline activity of Akt, but not that of NF-kB, was significantly higher in IGHV UM than M cells (*p* ≤0.001) (Figure 6A, 6B). The same results were confirmed by evaluating the expression of the active phosphorylated form of Akt and the nuclear translocation of the NF-kB components p50 and p65 by western blot analyses (Supplementary Figure S8A, S8B).

Akt activity was further upregulated by the presence of M2–10B4 SCs (Figure 5C and Supplementary Figure S9A), whereas NF-kB activity was not, as previously reported [4]. ZA significantly increased Akt activity in IGHV UM cells (*p* ≤ 0.009), and this effect was particularly evident when ZA and SCs were used in combination (*p* ≤ 0.03) (Supplementary Figure S9A). Of note, ZA also increased NF-kB activity in IGHV UM cells, both in the absence and in the presence of SCs (*p* ≤ 0.05 and *p* ≤ 0.04, respectively) (Supplementary Figure S9B). Unlike ZA, SIM reduced the baseline Akt and NF-kB activities (*p* ≤ 0.02), and significantly abrogated the SC-induced up-regulation of Akt and NF-kB activity (*p* ≤ 0.03; Figure 6C, 6D), Akt phosphorylation (Supplementary Figure S8C) and NF-kB nuclear translocation (Supplementary Figure S8D).

### Specific inhibitors of ERK1–2, RhoA kinase and HIF-1α reverse the MDR phenotype of IGHV UM cells

We also tested the activity of specific inhibitors targeting ERK1–2 kinases (PD98059, PD), RhoA kinase (Y27632, Y276) and HIF-1α (YC-1) in IGHV UM cells cultured alone or in the presence of SCs. Of note, the three inhibitors, as well as the upstream Mev pathway inhibitor SIM, had no impact on SCs viability after 48 hours of culture and did not increase the cytotoxic activity of Doxo toward SCs (Supplementary Figure S10).

**Figure 7:**
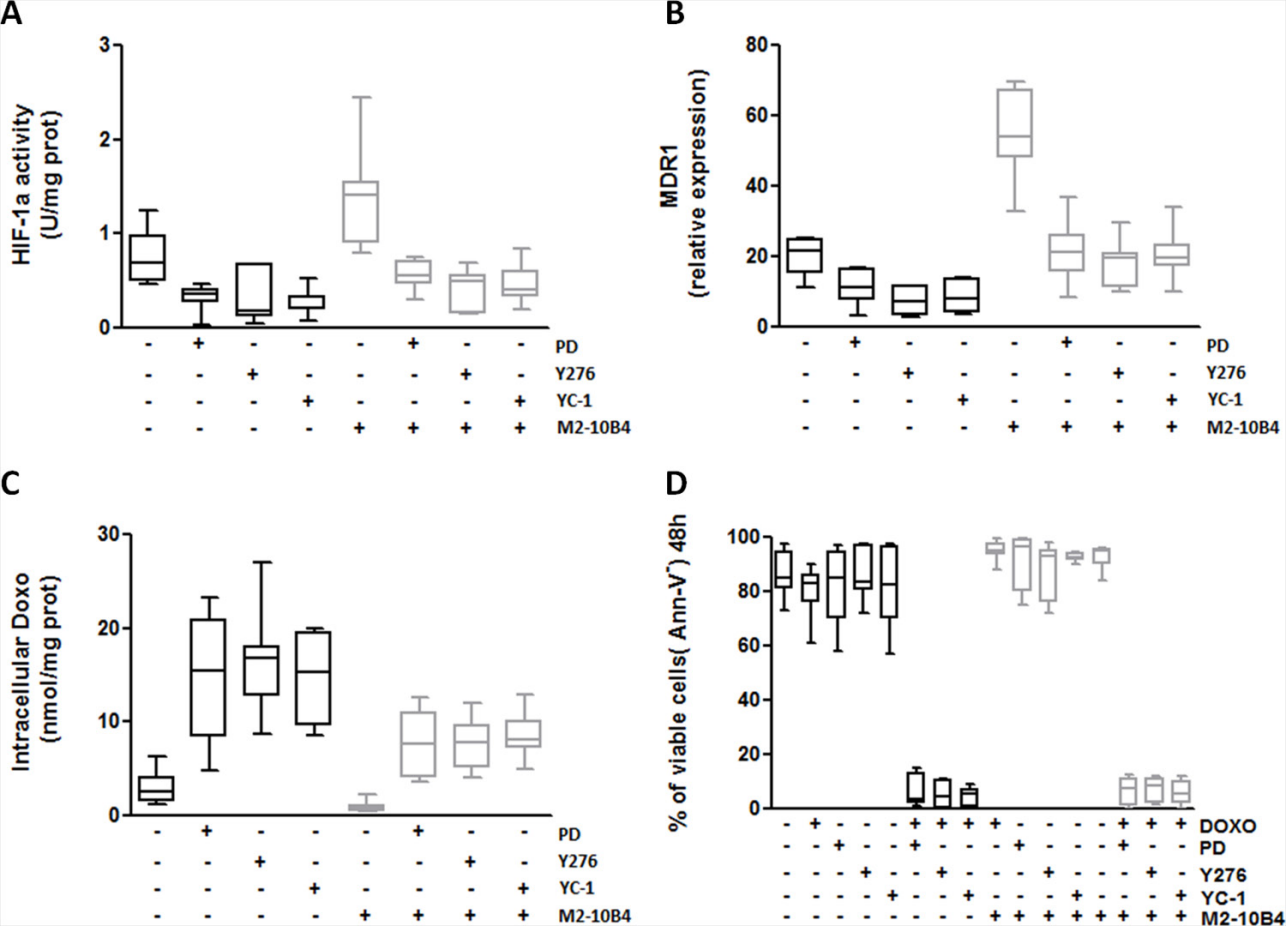
Specific inhibitors of RhoA, ERK1–2 and HIF-1α effectively reverse the MDR phenotype of IGHV UM cells IGHV UM cells were left untreated or treated with the ERK1–2 kinase inhibitor PD (10 μM), the RhoA kinase inhibitor Y276 (10 μM) and the HIF-1α inhibitor YC-1 (10 μM), both in the absence and in the presence of M2–10B4 SCs. **A.** HIF-1α activity. Co-culture with M2–10B4 SCs significantly increased the activity of HIF-1α (*p* = 0.0014). PD, Y276 and YC-1 significantly reduced the activity of HIF-1α in IGHV UM cells after 24 hours of culture, both in the absence (*p* always ≤ 0.0048) and in the presence (*p* always ≤ 0.0052) of SCs. **B.** MDR1 expression. Twenty four-hour co-culture with M2–10B4 SCs significantly increased the expression of MDR1 in IGHV UM cells (*p* < 0, 0001). After PD, Y276 and YC-1 treatment a significant decrease in MDR1 expression was observed in IGHV UM cells cultured alone (*p* always ≤ 0.001) and in the presence of SCs (*p* always ≤ 0.0001). **C.** Doxo intracellular accumulation. Intracellular Doxo was significantly lower in IGHV UM cells cultured with SCs than in IGHV UM cells cultured alone (*p* = 0.02). After 48-hour exposure to PD, Y276 and YC-1, Doxo accumulation was significantly increased, both in the absence (*p* always ≤ 0.003) and in the presence of M2–10B4 SCs (*p* always ≤ 0.0012). **D.** Percentage of CD19+/CD5+ viable cells. IGHV UM cells were exposed to 1 μM Doxo, used alone and in combination with each inhibitor (i.e. PD+Doxo, Y276+Doxo, YC-1+Doxo), both in the absence and in the presence of the M2–10B4 SCs. Cell viability was determined by Ann-V staining and cytofluorimetric analysis on CD19+/CD5+ cells after 48-hours of culture. Doxo alone, as well as the three inhibitors used as single agents, did not induce a decrease in cell viability. By contrast, the combinations PD+Doxo, Y276+Doxo and YC-1+Doxo significantly reduced the viability of IGHV UM cells both in the absence and in the presence (*p* always < 0.0001) of SCs. In panels A–D results are from 7 side-by-side experiments. Box and whiskers plot represent median values, first and third quartiles, and minimum and maximum values for each dataset.

In IGHV UM CLL cells, PD and Y276 markedly reduced HIF-1α phosphorylation (Supplementary Figure S11). Moreover, PD, Y276 and YC-1 reduced HIF-1α activity (Figure 7A) and MDR1 expression (Figure 7B), and increased intracellular Doxo accumulation (*p* always ≤ 0.0048) (Figure 7C) and Doxo-mediated cytotoxicity (*p* ≤ 0.0001) (Figure 7D and Supplementary Figure S12–S13) in IGHV UM cells, both in the absence and in the presence of SCs.

These results provided the proof-in-principle that inhibition of the Ras/ERK1–2/HIF-1α and RhoA/RhoA kinase/HIF-1α cascades, and the down-regulation of MDR1 gene expression and Pgp activity are mechanisms exploited by SIM to restore the chemosensitivity of MDR+ IGHV UM cells.

## DISCUSSION

This study shows that IGHV UM cells are endowed with a MDR phenotype which grants them a greater resistance to Doxo-induced cytotoxicity compared with IGHV M cells. Mechanistic insights have shown that IGHV UM cells, according to their accelerated Mev pathway activity [15], had significantly higher activity of the Mev-regulated Ras/ERK1–2 and RhoA/RhoA kinase signaling cascades, increased HIF-1α phosphorylation and activity, higher MDR1 gene expression, very effective Doxo extrusion and enhanced survival compared with IGHV M cells. The MDR signature of IGHV UM cells is at least partially dependent on BCR signaling, as shown by the inhibitory effect exerted by ibrutinib on the Mev pathway and Mev-regulated signaling.

The constitutive MDR features of IGHV UM cells are further exacerbated by the incubation with SCs, which stimulates the Mev pathway and its downstream signaling cascades. Mev pathway manipulation with SIM, and specific ERK1–2, RhoA kinase or HIF-1α inhibitors reset the chemosensitivity of IGHV UM cells to the same levels of IGHV M cells, and neutralize the MDR-enhancing effect operated by SCs.

A role for MDR1 in CLL chemoresistance has previously been suggested by the association of Pgp expression with IGHV UM genes and poor prognosis cytogenetics [23]. Our data confirm that the MDR1 gene, which is a primary transcriptional target of HIF-1α [24, 25], is constitutively more expressed in IGHV UM than in M cells. HIF-1 is a heterodimeric transcription factor composed of an oxygen-induced HIF-1α and a constitutively expressed HIF-1β subunit. During hypoxia, HIF-1α is positively regulated by the activation of the RhoA-dependent signaling cascade [16]. Under non-hypoxic conditions, the expression and transcriptional activity of HIF-1α are regulated by growth factors and cytokines through the activation of kinase pathways, such as the Ras/ERK1–2 and the PI3k/Akt pathways [17]. Unlike their normal counterparts, CLL cells express HIF-1α even under normoxia [26], and its transcriptional activity regulates CLL cells survival by inducing the expression of growth factors, such as VEGF, and the cytokines macrophage migration-inhibition factor (MIF) and midkine [26–28]. This is the first report showing that HIF-1α activity is significantly higher in IGHV UM than in M cells, due to the higher activity of the upstream Ras/ERK1–2 and RhoA/RhoA kinase signaling cascades in these cells. The MDR signature of IGHV UM cells is partially dependent on their tonic BCR signaling, as shown by the inhibitory effect exerted by ibrutinib but the lack of stimulatory effect exerted by anti-IgM.

Differently from anti-IgM-mediated BCR stimulation, SCs exposure further increase the activity of Ras/ERK1–2 and RhoA/RhoA kinase signaling pathways and HIF-1α in IGHV UM cells. As a result, the target gene MDR1 is over-expressed, Doxo is more efficiently extruded and IGHV UM cells are further protected from Doxo-induced cytotoxicity. SCs are known to confer chemoresistance to CLL cells. One of the main players of the SC-induced MDR is the CXCL12/CXCR4 axis, which is known to activate the signal transducers ERK1–2 and Akt, and trigger LFA1 affinity to the integrin ICAM-1, through a RhoA-mediated mechanism [29]. It is already known that the BCR signaling modulates the activity of the CXCL12/CXCR4 axis, in fact the blockade of the BCR signaling with a BTK inhibitor decreases the migration of CXCR4-expressing CLL cells toward CXCL12 produced by SCs [30].

The Mev pathway and the CXCL12/CXCR4 axis may reciprocally affect their activity, as shown by at least two observations: 1) for optimal signaling CXCR4 must be incorporated into membrane lipid rafts, whose formation require membrane cholesterol, and which orchestrate the interaction of the small GTPases Rac and Rho with their downstream transducers [31]; 2) hypercholesterolemia induces the secretion of CXCL12 and drives the migration of CD19+/CXCR4+ B lymphocytes from the bone marrow to the peripheral blood by interfering with the CXCL12/CXCR4 axis [32]. Further studies aiming at elucidating the connections between the Mev pathway and the CXCL12/CXCR4 axis in CLL are currently ongoing in our laboratory.

Statins and aminobisphosphonates, by targeting key enzymes in the Mev pathway, cause the intracellular deprivation of isoprenoid moieties such as FPP and GGPP, thus preventing Ras and RhoA prenylation and the activation of their downstream signaling pathways [33]. We have recently reported that ZA effectively interrupts Ras- and RhoA-dependent downstream signalling pathways, abrogates Pgp expression, and restores Doxo-induced cytotoxicity in MDR+ human cancer cell lines derived from solid tumors [14]. In CLL, ZA was not an ideal chemosensitizing agent since it inhibited Ras/ERK1–2 and RhoA/RhoA kinase signaling pathways, HIF-1α activity, and MDR1 expression, but did not increase Doxo-induced cytotoxicity. By contrast, SIM was effective in reversing the resistance of IGHV UM cells to Doxo, also in the presence of the protective effect exerted by SCs. This different efficacy was due to a stronger ability of SIM to increase the intracellular amount of Doxo, especially in the presence of SCs, but also to a differential regulatory effect exerted by the two compounds on the pro-survival factors Akt and NF-kB. We have already reported that ZA induces the activation of NF-kB in tumor-associated macrophages, reverting their phenotype from tumor-permissive into tumoricidal cells [34]. A similar upregulation of the Akt/NF-kB pathway was observed in IGHV UM cells after ZA, but not after SIM exposure. This at least partially accounted for a ZA-mediated pro-survival activity which most likely prevailed on the death signals triggered by Doxo.

Among the chemo-sensitizing agents that we have tested, statins are the best candidate for clinical translation, since they are commonly used in the clinical practice as cholesterol-lowering agents. Previous data have shown that SIM, although potently effective in blocking the proliferation and inducing apoptosis of CLL cells *in vitro* [35–37], is not capable of significantly reducing the tumor burden when administered as a single agent in previously untreated CLL patients [37]. By contrast, statins effectively increase the susceptibility of both drug-sensitive and drug-resistant CLL and lymphoma cells to dexamethasone and cytotoxic drugs [38], even synergizing with purine analogs to induce apoptosis of CLL cells [35]. Schmidmaier et al. have already reported that the Mev pathway and the downstream RhoA/RhoA kinase signaling pathway mediate SC-induced MDR, and that targeting of this pathway by SIM may improve the efficacy of anti-myeloma therapies by reduction of SC-induced MDR [39]. The same authors showed in a phase II clinical trial that SIM could overcome MDR in refractory myeloma receiving chemotherapy with bortezomib or bendamustine [40]. In the CLL setting, Chae et al. recently reported data on a retrospective analysis showing that the use of statin and aspirin is associated with improved outcome in CLL patients receiving salvage fludarabine, cyclophosphamide and rituximab (FCR) chemotherapy [41], providing the rationale for a prospective study aimed at evaluating the effects of statins in CLL patients receiving chemoimmunotherapy. Since none of the drugs included in the FCR regimen are substrates of the Pgp, further studies aimed at determining the role and mechanism of action of statins in chemo-sensitizing CLL cells are warranted.

Doxo accumulation as a read-out assay to test Pgp activity could be considered a limitation of our study, due to previous data showing that the addition of anthracyclines to alkylating agents did not improve the clinical outcome of CLL patients [8]. More recently, however, the addition of mitoxantrone or epirubicine to fludarabine-based regimens has been shown to improve response rates [42–44]. Skribek et al. have reported that anthracyclines can have direct cytotoxic effects on CLL cells *in vitro* irrespectively to age, clinical stage or cytogenetic markers like del(17p) [45]. These observations and our data are strong incentives to reconsider the therapeutic potential of anthracyclines in CLL, especially when used in combination with chemo-sensitizing agents targeting the Mev pathway such as statins, to treat specific subsets of high-risk CLL patients (e.g. IGHV UM patients). Moreover, the chemo-sensitizing effects that we observed by targeting ERK1–2 and RhoA kinase, as well as the transcription factor HIF-1α, in addition to providing a confirmatory evidence about the mechanistic role played by these pathways in the MDR+ phenotype of IGHV UM cells, opens new perspectives on innovative targets amenable to therapeutic interventions.

Interestingly, recent data have shown that Pgp may be involved in the decreased anti-cancer efficiency and modified pharmacological properties of novel targeted agents, such as imatinib [46] and second-generation tyrosine kinase inhibitors [47]. Therefore, the study of Pgp activity and its modulation might have a broad impact in the current era of targeted therapies, providing important information on the pharmacokinetic and the anti-cancer effects of these novel agents.

## MATERIALS AND METHODS

### Patients

Peripheral blood (PB) samples were collected from 63 untreated CLL patients between March 2008 and November 2012 after informed consent. Patient demographics and clinical characteristics are reported in Supplementary Table S1. CLL was defined by clinical examination, PB cell count and immunophenotypic criteria. Tumor IGHV rearrangements were amplified starting from genomic DNA and sequences with deviations of < 2% or ≥ 2% from the germline IGHV sequence were considered IGHV UM or M as previously reported [48].

### Cell purification and cell lines

Peripheral blood mononuclear cells (PBMC) from IGHV M and UM patients were isolated from fresh samples by density gradient centrifugation (Ficoll-Histopaque, PAA Laboratories GmbH, Linz, Austria). PBMC were used without further manipulation when they contained more than 90% of CLL cells (CD19+/CD5+). When CLL cells were ≤ 90% they were purified by negative selection using a Magnetic Bead-Activated Cell Sorting (MACS^®^) with a B Cell Isolation Kit II (Miltenyi Biotec, Bologna, Italy).

In selected experiments, the murine marrow-derived stromal cell line M2–10B4 (ATCC # CRL-1972) was used.

### Chemicals

Electrophoresis reagents were obtained from Bio-Rad Laboratories (Hercules, CA, USA). The protein content of cell lysates was assessed with the bicinchoninic acid kit from Sigma Chemical Co (St. Louis, MO, USA). ZA was a kind gift from Novartis (Basel, Switzerland). SIM and Y276 were purchased from Calbiochem (San Diego, CA, USA). When not otherwise specified, all the other reagents were purchased from Sigma Chemical Co.

### Culture and co-culture conditions

CLL cells were cultured in 24-well plates (Costar, Cambridge, MA, USA) (10^6^ cells/well) at 37°C in a 5% CO_2_ humidified incubator. The standard culture medium was RPMI 1640 (Euroclone, Milano, Italy) supplemented with 10% FCS (Euroclone), 2 mM L-glutamine, 100 U/ml penicillin and 100 μg/ml streptomycin.

For co-culture experiments, the M2–10B4 murine SCs were harvested through Trypsin-EDTA (Sigma-Aldrich, Milano, Italy) digestion and plated (150 × 10^3^ cells/well) in complete culture medium for 24 hours. CLL cells (1 × 10^6^ cells/well) were added to the culture the following day. In selected experiments, IGHV M and UM CLL cells were exposed to ZA 1 μM, SIM 1 μM, ERK1–2 kinase inhibitor PD 10 μM, HIF-1α inhibitor YC-1 10 μM, RhoA kinase inhibitor Y276 10 μM, in the absence and in the presence of Doxo 1 μM. In selected experiments, CLL cells (5 × 10^6^ cells/well) were pre-incubated in complete RPMI medium with or without ibrutinib (1 μM) for 30 minutes at 37°C, and then stimulated for 24 hours with 10 μg/well anti-IgM polyclonal goat F(ab’)_2_ (Southern Biotechnologies Birmingham, AL, USA) immobilized on 24-wells plates.

### Mev pathway activity

After 24 hours of culture, 1 × 10^6^ CLL cells were incubated for another 24 hours with 1 μCi of [^3^H]acetate (3600 mCi/mmol; Amersham International, Piscataway, NJ, USA) to measure the intracellular synthesis of cholesterol and FPP. Lipids were extracted in methanol/hexane, resolved by thin layer chromatography and quantified by liquid scintillation counting as reported in [15]. According to the titration curve, the results are expressed as fmol/1 × 10^6^ cells.

### Ras and RhoA activity

To evaluate Ras and RhoA activity, their GTP-bound fraction, taken as an index of the G-protein activation [49], was measured after 24 hours of culture. Ras-GTP was detected in a pull-down assay as previously reported [50]; RhoA-GTP binding was measured with the G-LISA RhoA Activation Assay Biochem Kit (Cytoskeleton Inc, Denver, CO, USA), according to the manufacturer's instructions. For each set of experiments a titration curve was prepared using serial dilution of the RhoA-GTP positive control of the kit. Data are expressed as U absorbance/mg cell proteins (U mg/prot).

### RhoA kinase activity

RhoA kinase activity was measured after 24 hours of culture on 2 × 10^6^ cells with the CycLex Rho Kinase Assay Kit (CycLex Co., Nagano, Japan), as already described [50]. For each set of experiments a titration curve was set using serial dilution of recombinant RhoA kinase (Rock2, MBL Inc., Woburn, MA, USA). Data are expressed as U absorbance/mg cell proteins (U mg/prot).

### Western blot (WB)

After 24 hours of culture 2 × 10^6^ cells were lysed in MLB buffer (125 mM Tris-HCl, 750 mM NaCl, 1% v/v NP40, 10% v/v glycerol, 50 mM MgCl_2_, 5 mM EDTA, 25 mM NaF, 1 mM NaVO_4_, 10 μg/ml leupeptin, 10 μg/ml pepstatin, 10 μg/ml aprotinin, 1 mmol/L phenylmethylsulfonyl fluoride, pH 7.5), sonicated (with two bursts of 10 s; Labsonic sonicator, Sartorius Stedim Biotech S.A., Aubagne Cedex, France), and centrifuged at 13000 × g for 10 min at 4°C. Ten μg cell lysates were subjected to SDS-PAGE, transferred to polyvinylidene fluoride membrane sheets (Immobilon-P, Millipore, Billerica, MA, USA) and probed with the following antibodies: anti phospho-(Thr202/Tyr204, Thr185/Tyr187)-ERK1–2 (Millipore); anti-ERK1–2 (Millipore); anti-phospho-(Ser473)-Akt (Millipore); anti-Akt (Millipore); anti- glyceraldehyde-3-phosphate dehydrogenase (GAPDH, used as control of equal protein loading; Santa Cruz Biotechnology Inc., Santa Cruz, CA, USA), followed by the secondary peroxidase-conjugated antibodies (Bio-Rad). Proteins were detected by enhanced chemiluminescence (PerkinElmer, Waltham, MA, USA).

### HIF-1α activity

Nuclear proteins from 2 × 10^6^ cells were extracted after 24-hour culture using the Nuclear Extract Kit (Active Motif, Rixensart, Belgium), and quantified. The activity of HIF-1α was assessed on 10 μg nuclear extracts using the TransAM™ HIF-1α Transcription Factor Assay Kit (Active Motif), according to manufacturer's instructions. To assess the procedure specificity, a competition assay was performed by adding an excess (20 pmol) of HIF-1α consensus oligonucleotide to nuclear extracts derived from CLL cells. Data are expressed as U absorbance/mg cell proteins (U mg/prot).

### Real-time polymerase chain reaction (RT-PCR)

After 24 hours of *ex vivo* culture, total RNA was extracted from 1 × 10^6^ cells and reverse-transcribed using the QuantiTect Reverse Transcription Kit (Qiagen, Hilden, Germany). RT-PCR was carried out using IQ^−^ SYBR Green Supermix (Bio-Rad), according to the manufacturer's instructions. The sequences of MDR1 primers were 5′-TGCTGGAGCGG TTCTACG-3′, 5′-ATAGGCAATGTTCTCAGCAATG-3′. The sequences of GAPDH primers were 5′-GA AGGTGAAGGTCGGAGT-3′, 5′-CATGGTGGAAT CATATTGGAA-3′. The relative expression of each sample was performed comparing the MDR1 PCR product with the *GAPDH* product, used as a housekeeping gene, with the Bio-Rad Software Gene Expression Quantitation (Bio-Rad).

### NF-kB and Akt activity

NF-kB activity was measured on 10 μg nuclear extracts using the TransAM Flexi NF-kB Family assay kit (Active Motif), according to the manufacturer's instructions. To assess the procedure specificity, a competition assay was performed by adding an excess (20 pmol) of the probe containing the NF-kB consensus sequence to the positive control nuclear extracts provided by the kit.

Akt activity was measured on 2 × 10^6^ cells with the CycLex Akt/PKB Kinase Assay/Inhibitor screening kit (CycLex Co.) in 96-well plates pre-coated with the Akt substrate AkTide-2T, according to the manufacturer's instructions. The titration curve was prepared using serial dilutions of recombinant Akt (CycLex Co.).

NF-kB and Akt activity are expressed as U absorbance/mg cell proteins (U mg/prot).

### Intracellular doxorubicin accumulation

To evaluate the activity of Pgp the intracellular accumulation of the Pgp substrate Doxo was measured. To this aim, 1 × 10^6^ cells were incubated with 1 μM Doxo cultured alone or in presence of inhibitors for 48 hours; intracellular Doxo was measured spectrofluorimetrically, at excitation and emission wavelengths of 475 and 553 nm, as reported earlier [50]. A blank was prepared in the absence of cells in each set of experiments, and its fluorescence was subtracted from that measured in each sample. Fluorescence was converted in nmol Doxo/mg cell proteins (nmol/mg prot) using a previously prepared calibration curve.

### Quantification of apoptotic and viable cells

After 48 hours of culture, cells were harvested, washed in PBS and stained with anti-CD19 PerCP (Beckman Coulter, Milano, Italy) and anti-CD5 APC (Dako SpA, Milano, Italy) monoclonal antibodies. The percentage of apoptotic cells was determined by Annexin-V (Ann-V) staining with the MEBCYTO-Apoptosis Kit (MBL Medical and Biological Laboratories, Naka-ku Nagoya, Japan). Flow cytometry was performed with a FACSCalibur and CELLQuest software (Becton Dickinson, Mountain View, CA, USA).

### Statistical analysis

Statistical analysis was performed with the SigmaStat 3.5 software (Systat Software Inc., Chicago, IL, USA) and with GraphPad Prism version 5.01 for Windows (GraphPad Software, San Diego, CA, USA). All datasets were evaluated with a normality test: data with Gaussian or approximately Gaussian distribution were compared by *t*-test, in all other cases a Mann-Whitney rank sum test was used. When experiments involved different culture conditions of the same sample a repeated-measures analysis was done.

Results are expressed as mean ± SEM, unless otherwise specified.

Statistical significance was defined as *p* value < 0.05.

## SUPPLEMENTARY MATERIALS AND METHODS FIGURES AND TABLE


