# Supplementary material for: Simvastatin and downstream inhibitors circumvent constitutive and stromal cell-induced resistance to doxorubicin in IGHV unmutated CLL cells
Source: Oncotarget. 2015 May 27;6(30):29833–46. doi: 10.18632/oncotarget.4006 (PMC4745766; doi:10.18632/oncotarget.4006)
Supplement: Supplementary file 1 [file oncotarget-06-29833-s001.pdf]

MATERIALS AND METHODS

HIF-1α phosphorylation

To assess HIF-1α phosphorylation the whole cell lysate was immunoprecipitated with a polyclonal anti-HIF-1α antibody (Santa Cruz Biotechnology Inc., Santa Cruz, CA, USA), then probed with a biotin-conjugated anti-phosphoserine antibody (Sigma Chemical Co., St. Louis, MO, USA) and subjected to a streptavidin-horseradish peroxidase-conjugated polymer (Sigma Chemical Co.).

NF-kB nuclear translocation

Nuclear proteins from 2×10^6 cells were extracted after 24-hour culture using the Nuclear Extract Kit (Active Motif, Rixensart, Belgium), and quantified. Ten µg nuclear extracts were probed with the following antibodies: anti-p50 (Santa Cruz Biotechnology Inc.), anti-p65 (Santa Cruz Biotechnology Inc.), anti-TATA box binding protein (TBP, used as control of equal protein loading; Santa Cruz Biotechnology Inc.), followed by the secondary peroxidase-conjugated antibodies (Bio-Rad, Hercules, CA, USA). Proteins were detected by enhanced chemiluminescence (PerkinElmer, Waltham, MA, USA).

SUPPLEMENTARY FIGURES AND TABLE

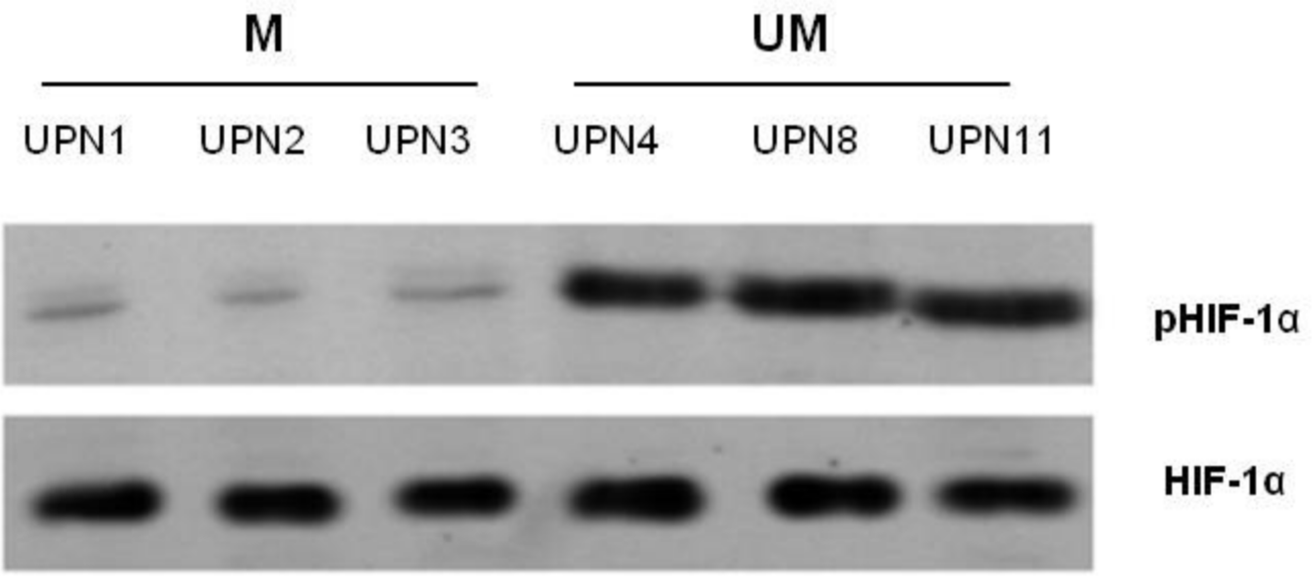

Supplementary Figure S1: Phosphorylation of HIF-1α in IGHV M and UM CLL cells. HIF-1α phosphorylated on serine (pHIF-1α) and total HIF-1α expression in IGHV M and UM cells were measured by Western blot analysis. IGHV UM cells had a higher baseline expression of pHIF-1α than M cells (UPN, unique patient number). Results are from three representative experiments performed in each group of patients.

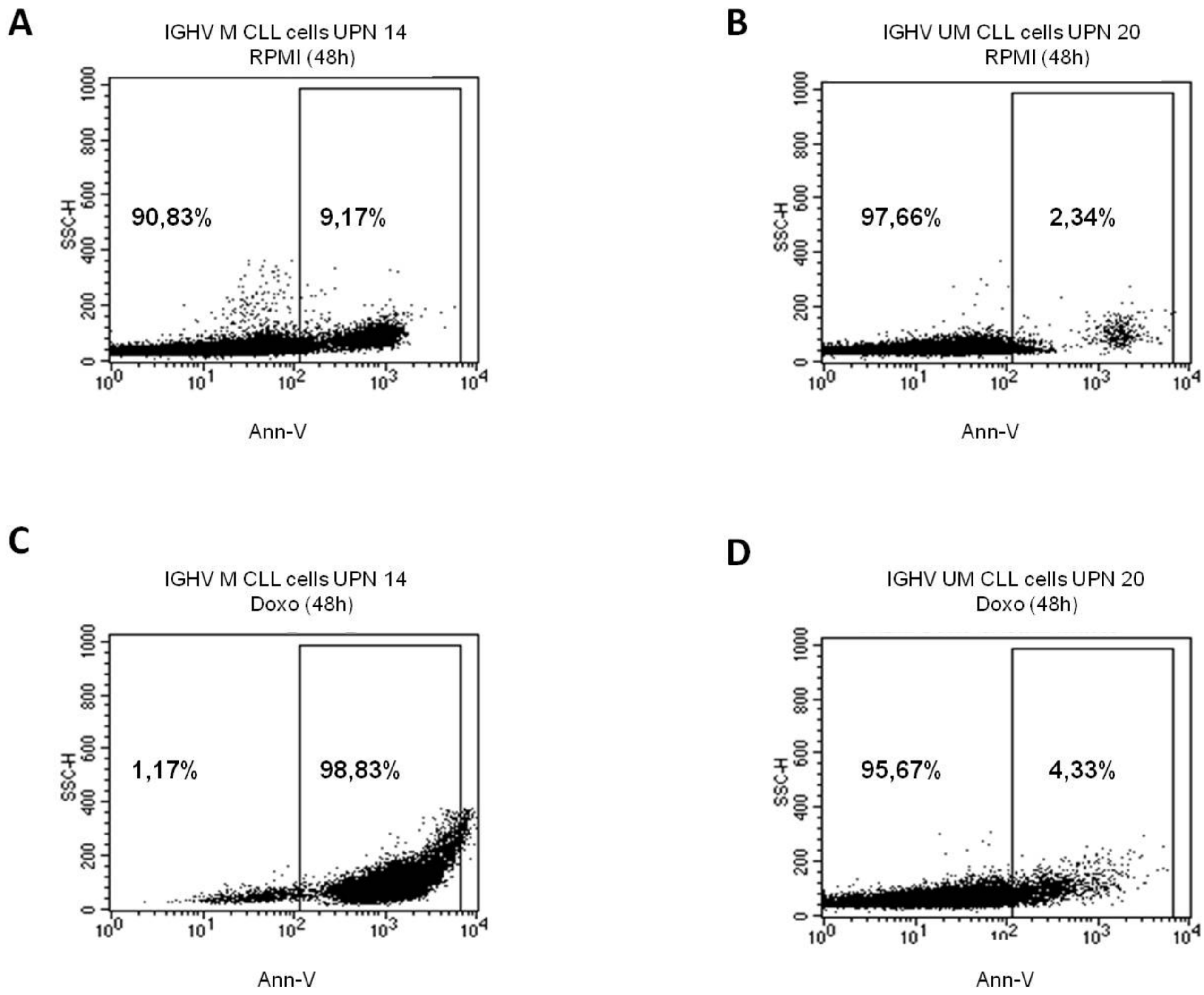

Supplementary Figure S2: Viability of IGHV M and UM CLL cells treated with Doxo. Representative analyses of Ann-V expression gating on CD19+/CD5+ IGHV M and UM cells (UPN, unique patient number) after 48-hour exposure to RPMI **A, B.** or Doxo **C, D.** Doxo induced a significant decrease in the viability of IGHV M but not UM cells.

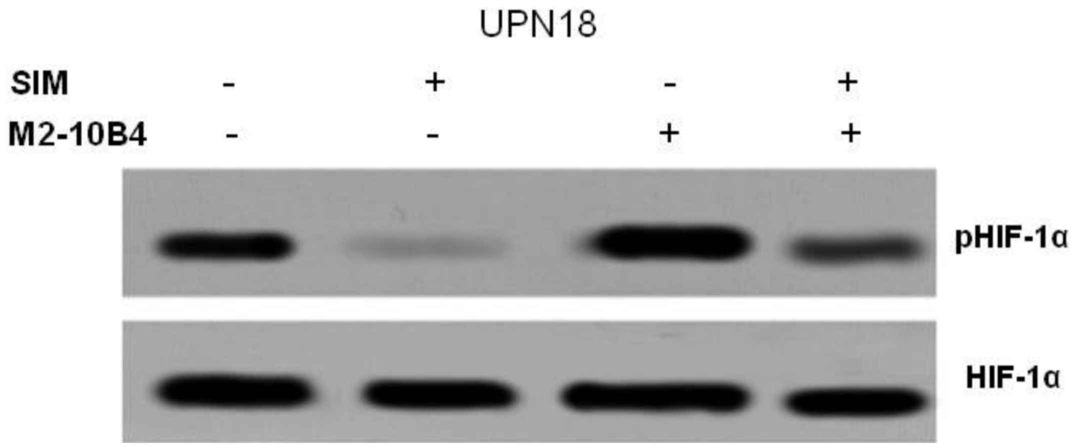

Supplementary Figure S3: SIM-mediated increase of HIF-1α phosphorylation in IGHV UM cells. IGHV UM cells, cultured alone or in the presence of the murine stromal cell line M2–10B4, were exposed to 1 µM SIM. Co-culture with M2–10B4 SCs increased the phosphorylation of HIF-1α. The activity of HIF-1α was reduced by SIM, both in the absence and presence of M2–10B4 SCs. Assessment of HIF-1α phosphorylation was performed as reported in Materials and Methods. Results are from one representative experiment.

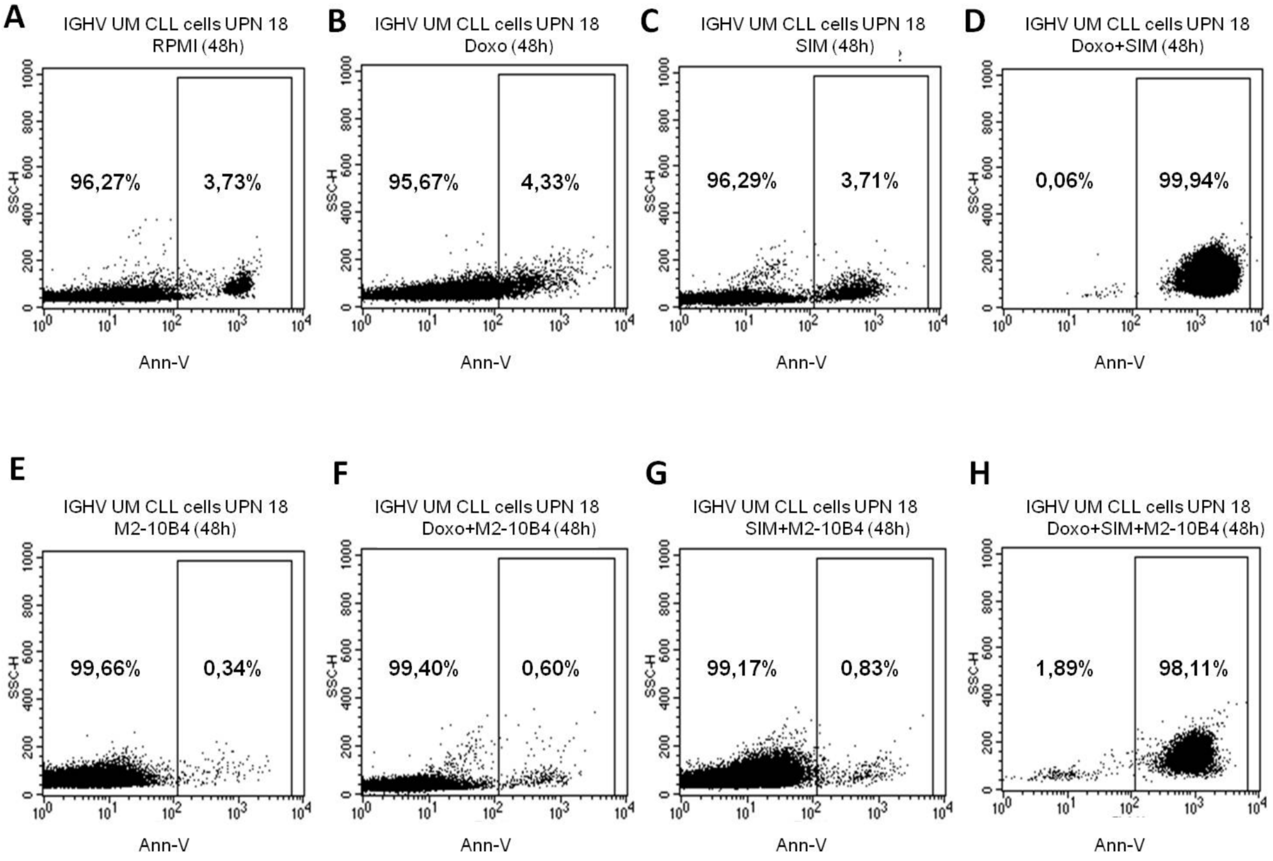

Supplementary Figure S4: Viability of IGHV UM CLL cells treated with Doxo, SIM and Doxo+SIM. Representative analyses (UPN, unique patient number) of Ann-V expression gating on CD19+/CD5+ IGHV UM cells exposed for 48 hours to RPMI, Doxo, SIM, and the combination SIM+Doxo, in the absence **A–D.** and in the presence **E–H.** of the murine stromal cell line M2–10B4. Viability of IGHV UM cells was unaffected by SIM and Doxo used as single agents, whereas it was markedly decreased by the SIM+Doxo combination (D), also when CLL cells were co-cultured with the M2–10B4 SCs (H).

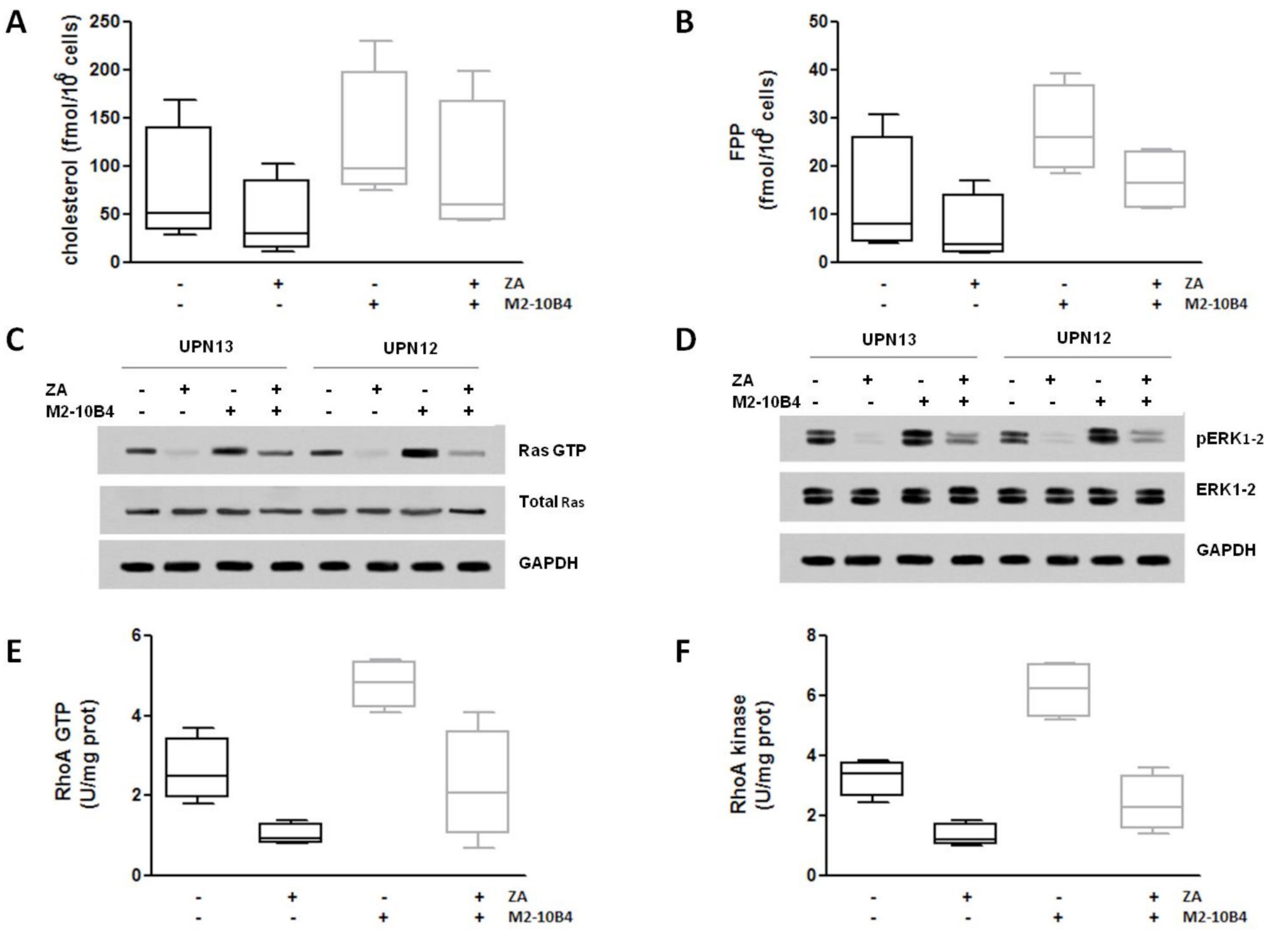

Supplementary Figure S5: ZA-mediated inhibition of Ras/ERK1–2 and RhoA/RhoA kinase signaling pathways. IGHV UM cells, cultured alone or in the presence of the murine stromal cell line M2–10B4, were exposed to 1 μM ZA. **A.** Cholesterol production. Co-culture with M2–10B4 SCs increased the levels of cholesterol in IGHV UM cells after 24 hours. Cholesterol production was inhibited by ZA in the absence (p = 0.05) and in the presence of SCs (p = 0.009). **B.** FPP production. Co-culture with M2–10B4 SCs increased the levels of FPP in IGHV UM cells (p = 0.006) after 24 hours of culture. FPP production was significantly inhibited by ZA in the absence (p = 0.05) and in the presence of SCs (p = 0.03). **C, D.** Ras and ERK1–2 kinase activity. Twenty four-hour co-culture with M2–10B4 SCs increased the expression of the active forms of Ras (Ras-GTP) and ERK1–2 kinase (pERK1–2) in IGHV UM cells. ZA reduced the expression of Ras-GTP (C) and pERK1–2 (D) in IGHV UM cells, both in the absence and in the presence of SCs. The results are from two representative experiments (UPN, unique patient number). **E, F.** RhoA-GTP and Rho Kinase activity. Twenty four-hour co-culture with M2–10B4 SCs significantly increased the levels of expression of RhoA-GTP and RhoA Kinase (p = 0.01 and p = 0.003, respectively). ZA reduced the expression of RhoA-GTP and RhoA Kinase in IGHV UM cells both in the absence (p = 0.03 and p = 0.004, respectively) and in the presence (p = 0.003 and p = 0.001, respectively) of SCs. Box and whiskers plot represent median values, first and third quartiles, and minimum and maximum values for each dataset (A, B and E, F).

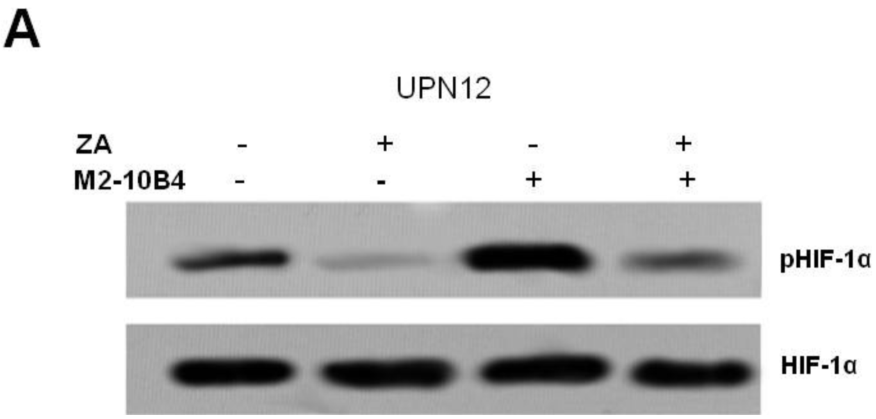

Supplementary Figure S6: ZA-mediated increase of HIF-1α phosphorylation in IGHV UM cells. IGHV UM cells, cultured alone or in the presence of the murine stromal cell line M2–10B4, were exposed to 1 μM ZA. Co-culture with M2–10B4 SCs increased the phosphorylation of HIF-1α (pHIF-1α). The expression of pHIF-1α was reduced by ZA, both in the absence and presence of M2–10B4 SCs. Assessment of HIF-1α phosphorylation was performed as reported in Materials and Methods. Results are from one representative experiment.

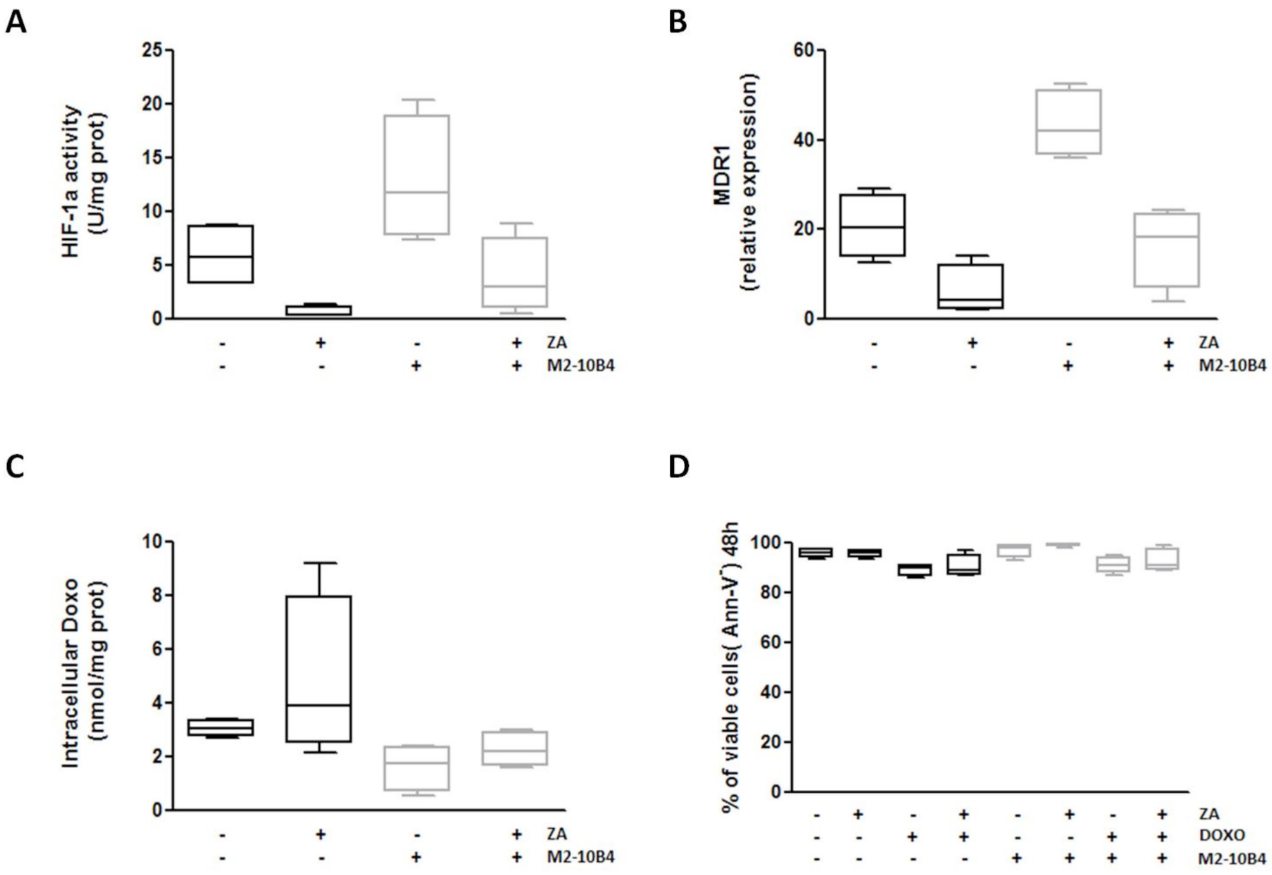

Supplementary Figure S7: ZA is effective in inhibiting HIF-1α activity and MDR1 gene expression, but not in sensitizing IGHV UM cells to Doxo-induced cytotoxicity. A, B. HIF-1α activity and MDR1 expression. Twenty four-hour co-culture with M2–10B4 SCs significantly increased the activity of HIF-1α ($p = 0.03$) and the expression of MDR1 ($p = 0.01$) in IGHV UM cells. HIF-1α activity and MDR1 expression were significantly reduced by ZA, both in the absence ($p = 0.05$ and $p = 0.01$, respectively) and in the presence ($p = 0.04$ and $p = 0.03$, respectively) of the murine stromal cell line M2–10B4. **C.** Intracellular Doxo accumulation. Co-culture of IGHV UM cells with M2–10B4 SCs reduced the levels of intracellular Doxo ($p = 0.01$). After 48 hours of culture, no increase in intracellular Doxo was observed in IGHV UM cells treated with ZA, both in the absence and presence of M2–10B4 SCs. **D.** Percentage of CD19+/CD5+ viable cells. IGHV UM cells were exposed to 1 μM Doxo, 1 μM ZA, and the combination ZA+Doxo for 48 hours, both in the absence and in the presence of SCs. Doxo and ZA+Doxo did not induce cell cytotoxicity, as shown by the high percentage of viable cells in all culture conditions. In all panels box and whiskers plot represent median values, first and third quartiles, and minimum and maximum values for each dataset.

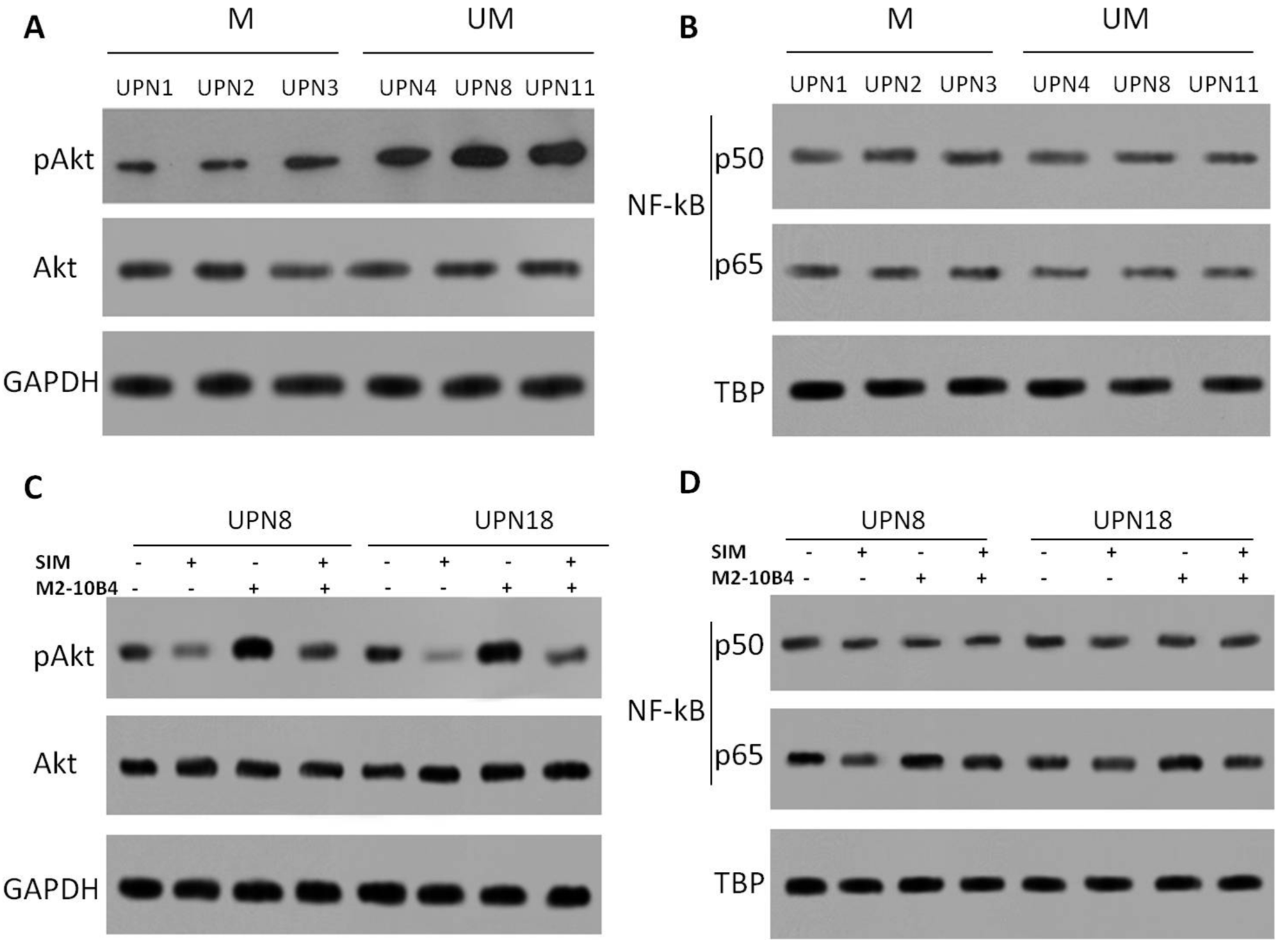

Supplementary Figure S8: Phosphorylation of Akt and nuclear translocation of NF-kB in CLL cells. Expression of Akt phosphorylated on serine 473 (pAkt) and total Akt in whole cells lysates **A, C.** and expression of the NF-kB components p50 and p65 in nuclear extracts **B, D.** of CLL cells, measured by Western blot analyses. (A) IGHV UM cells had higher baseline expression of pAkt than M cells (UPN, unique patient number). (B) By contrast, there was no difference in p50 and p65 expression between IGHV M and UM cells. Co-culture with M2–10B4 SCs increased the phosphorylation of Akt (C), but not the nuclear translocation of NF-kB (D). SIM markedly reduced Akt phosphorylation, both in the absence and presence of M2–10B4 SCs (C), whereas it only slightly reduced the nuclear levels of p50 and p65 (D). In panels A and B, results are from three representative experiments performed in each group of IGHV M and UM patients; in panels C and D results are from two representative experiments performed on IGHV UM cells.

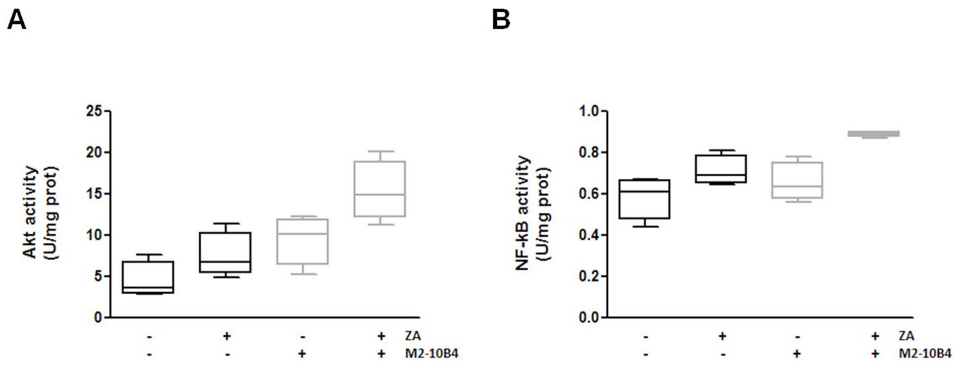

Supplementary Figure S9: Akt and NF-kB activity in CLL cells. A. Akt activity in IGHV UM cells was significantly upregulated by co-culture with M2–10B4 SCs ($p = 0.04$) and by exposure to ZA used alone ($p \leq 0.009$) or in the presence of M2–10B4 SCs ($p \leq 0.05$). **B.** NF-kB activity was unaffected by co-culture with M2–10B4 SCs, whereas it was significantly upregulated by ZA, both in the absence ($p \leq 0.05$) and in the presence ($p \leq 0.04$) of SCs. Box and whiskers plot represent median values, first and third quartiles, and minimum and maximum values for each dataset (A and B).

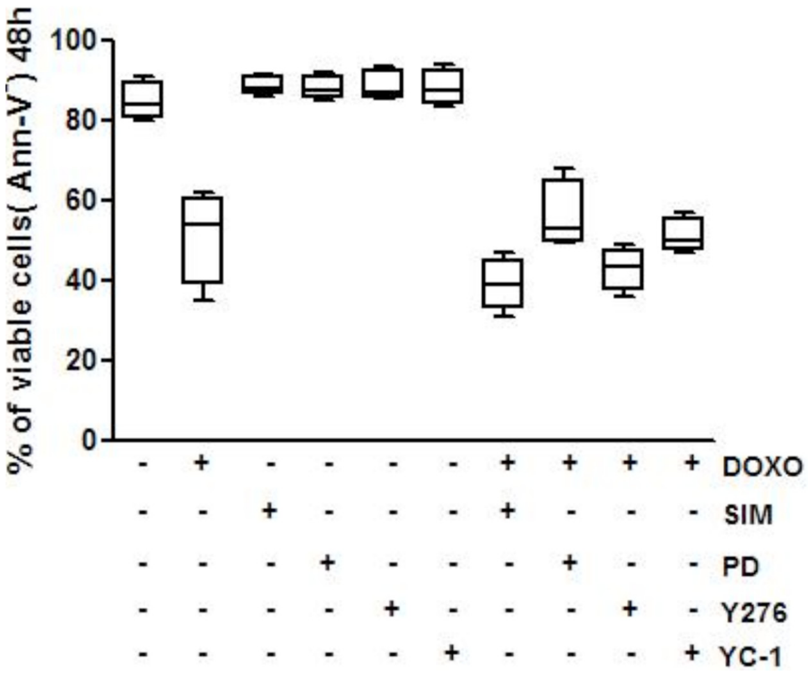

Supplementary Figure S10: SIM and specific inhibitors of RhoA kinase, ERK1–2 and HIF-1a do not affect M2–10B4 SCs viability when used alone or in combination with Doxo. M2–10B4 SCs were exposed to 1 µM SIM, 10 µM PD, 10 µM Y276 and 10 µM YC-1, used alone or in combination with Doxo, and evaluated for cell viability. Cell viability was determined by Ann-V staining and cytofluorimetric analysis after 48 hours of culture. Doxo significantly reduce M2–10B4 SCs viability ($p = 0.026$), whereas SIM, PD, Y276 and YC-1 did not reduce the M2–10B4 SCs viability when used as single agents. The combinations SIM+Doxo, PD+Doxo, Y276+Doxo and YC-1+Doxo did not increase the cytotoxic effect of single-agent Doxo toward SCs. Results are from 3 side by side independent experiments. Box and whiskers plot represent median values, first and third quartiles, and minimum and maximum values for each dataset.

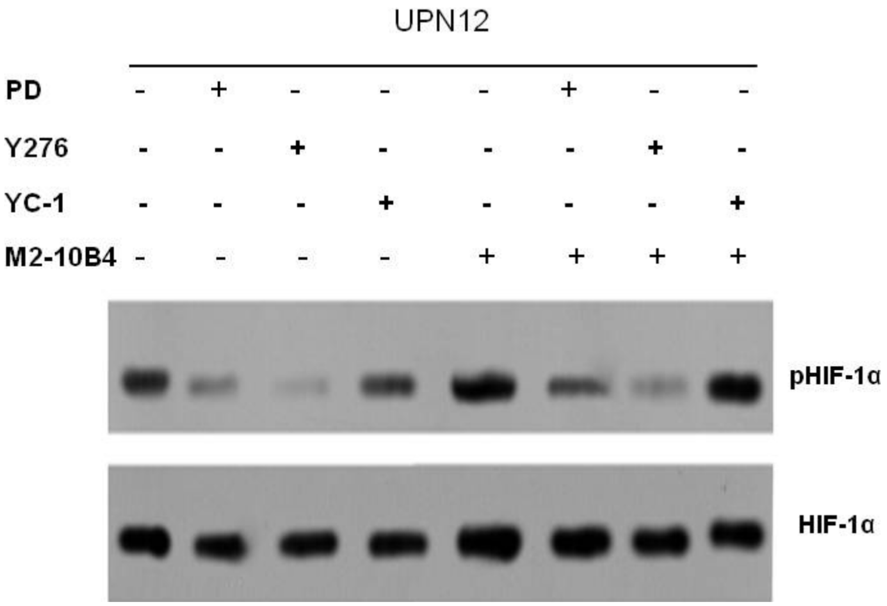

Supplementary Figure S11: Specific inhibitors of RhoA and ERK1–2 reduce HIF-1α phosphorylation. Co-culture of IGHV UM cells with M2–10B4 SCs significantly increased the phosphorylation of HIF-1α. The ERK1–2 inhibitor PD (10 μM) and the RhoA kinase inhibitor Y27632 (Y276) (10 μM) reduced the phosphorylation of HIF-1α (pHIF-1α) in IGHV UM cells, both in the absence and in the presence of M2–10B4 SCs. As expected, YC-1 reduced HIF-1α activity without affecting its phosphorylation.

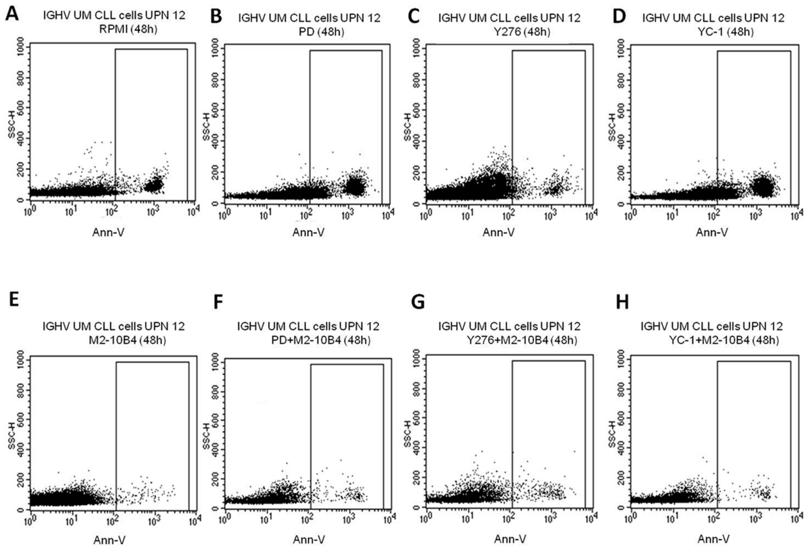

Supplementary Figure S12: Specific inhibitors of RhoA kinase, ERK1–2 and Hif-1a do not affect IGHV UM cells viability. Representative analyses (UPN, unique patient number) of Ann-V expression gating on CD19+/CD5+ IGHV UM cells exposed for 48 hours to RPMI, PD, Y276 and YC-1 in the absence **A–D.** and in the presence **E–H.** of M2–10B4 SCs. The 3 specific inhibitors did not exert a cytotoxic effect toward IGHV UM cells when used as single agents.

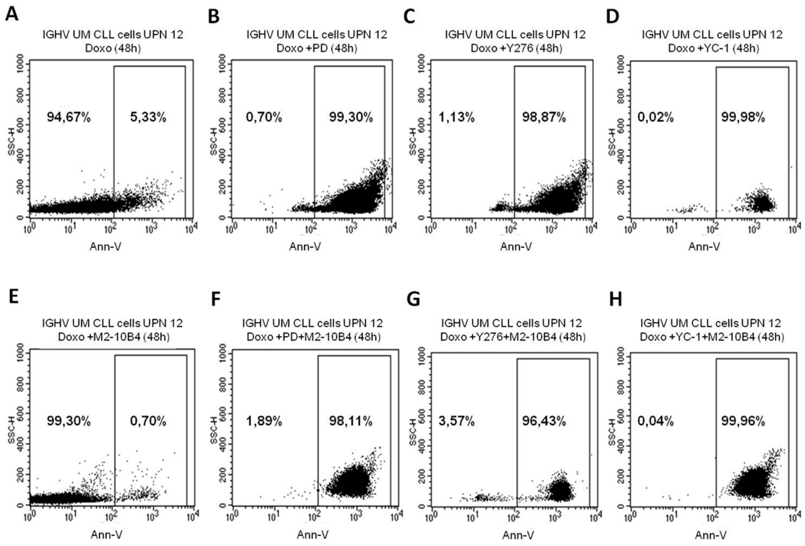

Supplementary Figure S13: Specific inhibitors of RhoA, ERK1–2 and HIF-1α effectively restore Doxo sensitivity of IGHV UM cells. Representative analyses (UPN, unique patient number) of Ann-V expression gating on CD19+/CD5+ IGHV UM cells exposed for 48 hours to 1 µM Doxo, used alone **A, B.** or in combination with specific inhibitors of RhoA kinase (PD), ERK1–2 (Y276) and HIF-1α (YC-1), both in the absence **B–D.** and in the presence **F–H.** of M2–10B4 SCs. Doxo did not induce a decrease in cell viability. By contrast, the combinations PD+Doxo, Y276+Doxo and YC-1+Doxo markedly reduced the viability of IGHV UM cells, both in the absence (B–D) and in the presence (F–H) of SCs.

Supplementary Table S1: Clinical and biological features of CLL patients

	All patients	UM patients
No. patients[1]	63	31 (49%)
Median age[2], years (range)	65 (43–82)	64 (45–81)
Female, no. (%)	23 (37%)	9 (29%)
Binet stage, no. (%)[3]		
A	49 (82%)	20 (69%)
B	8 (13%)	6 (21%)
C	3 (5%)	3 (10%)
Lymphocytes, #/µl[4,5]	15400 ± 1600	17900 ± 2800
Lymphocytes, %[4,6]	67.9 ± 2.0	67.0 ± 3.5
Hb, g/dl[4,5]	14.1 ± 0.2	13.8 ± 0.4
Plts, #/µl[4,6]	197000 ± 7100	194000 ±10800
LDH, U/ml[4,7]	359 ± 11	371 ± 13
β2M, g/dl[4,8]	2.1 ± 0.2	2.5 ± 0.4
% B CLL clone[4,9]	78.4 ± 2.0	79.7 ± 2.9
CD38+, no. (%)[2,10]	18 (28%)	13 (43%)
ZAP70+, no. (%)[2,11]	13 (20%)	9 (30%)
K light chain, no. (%)[5]	36 (58%)	17 (59%)
Cytogenetics[2]normal caryotype, no. (%)	25 (41%)	13 (45%)
Del (11), no. (%)	6 (12%)	3 (10%)
Del (13), no. (%)	25 (41%)	7 (24%)
Del (17), no. (%)	7 (11%)	3 (10%)

[1] All cases were evaluable for each of the variables, if not otherwise specified
[2] data available in 61 patients, 30 patients for UM category
[3] data available in 60 patients, 29 patients for UM category
[4] data expressed as mean ± SEM
[5] data available in 59 patients, 29 patients for UM category
[6] data available in 58 patients, 28 patients for UM category
[7] data available in 49 patients, 27 patients for UM category
[8] data available in 31 patients, 17 patients for UM category
[9] data available in 55 patients, 27 patients for UM category
[10] CD38 expression was considered positive when >30%
[11] ZAP70 expression was considered positive when >20%
